# Cell fate determining molecular switches and signaling pathways in Pax7-expressing somitic mesoderm

**DOI:** 10.1038/s41421-022-00407-0

**Published:** 2022-06-28

**Authors:** Cheuk Wang Fung, Shaopu Zhou, Han Zhu, Xiuqing Wei, Zhenguo Wu, Angela Ruohao Wu

**Affiliations:** 1 Division of Life Science, The Hong Kong University of Science and Technology, Clear Water Bay, Kowloon, Hong Kong SAR, China; 2 Department of Chemical and Biological Engineering, The Hong Kong University of Science and Technology, Clear Water Bay, Kowloon, Hong Kong SAR, China; 3 Present Address: Department of Pediatrics, University of California San Diego, San Diego, CA USA; 4 Present Address: Development, Aging and Regeneration Program, Sanford Burnham Prebys Medical Discovery Institute, La Jolla, CA USA

**Keywords:** Pluripotency, Genome-wide analysis of gene expression

## Abstract

During development, different cell types originate from a common progenitor at well-defined time points. Previous lineage-tracing of Pax7^+^ progenitors from the somitic mesoderm has established its developmental trajectory towards the dermis, brown adipocytes, and skeletal muscle in the dorsal trunk; yet the molecular switches and mechanisms guiding the differentiation into different lineages remain unknown. We performed lineage-tracing of Pax7-expressing cells in mouse embryos at E9.5 and profiled the transcriptomes of Pax7-progenies on E12.5, E14.5, and E16.5 at single-cell level. Analysis of single-cell transcriptomic data at multiple time points showed temporal-specific differentiation events toward muscle, dermis, and brown adipocyte, identified marker genes for putative progenitors and revealed transcription factors that could drive lineage-specific differentiation. We then utilized a combination of surface markers identified in the single-cell data, Pdgfra, Thy1, and Cd36, to enrich brown adipocytes, dermal fibroblasts, and progenitors specific for these two cell types at E14.5 and E16.5. These enriched cell populations were then used for further culture and functional assays in vitro, in which *Wnt5a* and *Rgcc* are shown to be important factors that could alter lineage decisions during embryogenesis. Notably, we found a bipotent progenitor population at E14.5, having lineage potentials towards both dermal fibroblasts and brown adipocytes. They were termed eFAPs (embryonic fibro/adipogenic progenitors) as they functionally resemble adult fibro/adipogenic progenitors. Overall, this study provides further understanding of the Pax7 lineage during embryonic development using a combination of lineage tracing with temporally sampled single-cell transcriptomics.

## Introduction

The spatiotemporal regulation of lineage progression of different tissues has always been a focus of developmental biology. Multiple lines of evidence have shown that different cell types branch out from a common progenitor at different time points^1–4^. These transient lineage specification events are hard to capture, making it extremely difficult to understand the key transcriptional regulators of these events. One example is the development of skeletal muscle and brown adipose tissue (BAT) from the dermomyotome, an epithelial structure in the dorsal part of segmented somites^5–7^. In rodents, both tissues are derived from the *Myf5*^+^ and *Pax7*^+^ mesodermal progenitors^8^. It is still unclear whether there are distinct subpopulations of cells within these common progenitors that give rise to different lineages, or whether the lineage specification occurs via transcriptional regulation of a multipotent precursor cell. To address this question, multiple lineage tracing experiments with stable genetically engineered reporter systems have been conducted to examine the fate of certain cell populations known to give rise to muscle and adipose tissue. Firstly, *Myf5* expressing mesodermal progenitors in *Myf5*^*Cre*^:*R26R3*^*YFP*^ mice were shown to give rise to muscles and BAT, but not white adipose tissue (WAT)^9^. Secondly, *Pax7*^+^ precursors are known to contribute to both the neural crest and the muscle tissue, with mouse *Pax7* expression found as early as E8.0 in the neural crest and later also in the somites^10^, and this lineage was also carefully examined with the *Pax7*^*Cre-ERT2*^ tamoxifen inducible system by Lepper and colleagues^11^: labeling of the *Pax7*^+^ cells at E8.5 or earlier resulted in reporter expression mainly in the neural crest, which was also observed by Murdoch et al.^10^; tamoxifen induction between E9.5-E10.5 led to labeling of multiple different tissues including muscles, BAT, and dorsal dermis; while late induction of recombination (i.e., after E12.5) predominantly labeled muscles^11^. A third study using a similar inducible lineage tracing system targeting the *En1* expressing cells showed that *En1*^*+*^ progenitors could also give rise to muscle, BAT and dermis^5^. These findings greatly advanced our knowledge of the muscle and BAT developmental origins, revealing unexpected heterogeneity of the *Myf5*^+^/*Pax7*^+^/*En1*^+^ precursors as well as the temporally dynamic changes in their potency.

Reprogramming experiments in fibroblasts by overexpression of master transcription factors, such as MyoD and Prdm16, demonstrated the roles of such transcription factors in establishing lineage-specific transcriptional programs of muscles and BATs respectively^12,13^. Strikingly, these self-reinforced lineages seem to be relatively plastic, and transitions between fate-established cells of different lineages were observed when important factors were targeted. In BAT, knockdown of Prdm16 shifted the lineage towards myoblasts^9^. In muscle, our previous work and the work from Wang et al. demonstrated that deletion of Pax7 in the juvenile myogenic precursor cells, or deletion of MyoD in myoblasts induced a cell fate change from myoblasts to brown adipocytes, although the change of cell fate in vivo is very inefficient postnatally^14,15^. Molecularly, we showed that Myf5 and MyoD, the known downstream targets of Pax7, induce the expression of a transcription repressor E2f4 that in turn suppresses the lineage of brown adipocytes by inhibiting the expression of Prdm16^14^. This Pax7-Myf5/Myod1-E2f4-Prdm16 axis also explained the sustained *Pax7* expression in muscle lineage, but not brown adipocytes^14^. In addition to the transcription factors, the muscle-enriched miR-133 also represses the BAT lineage in the muscles by directly targeting *Prdm16* mRNA^16^. Interestingly, overexpression of the Notch intracellular domain (NICD) in the *Pax7*-deficient muscle satellite cells repressed both MyoD and miR-133, leading to further shift to the BAT lineage^17^. Understanding the molecular regulation of the BAT/muscle/dermis lineage specification will help us understand the maintenance and plasticity of these cell types under adult homeostasis and pathological conditions, as well as provide potential therapeutic strategies for metabolic diseases. To further understand the complex regulation of the developmental process, systematic approaches at single-cell level should be employed.

Although the lineage-tracing experiments revealed the developmental origins of the muscle, BAT, and dorsal dermis, they cannot provide molecular explanations of the lineage specification. Moreover, manipulations of the key transcription factors were mainly done under in vitro culture systems, making it hard to elucidate the branching events in time and space during development. Single-cell RNA sequencing (scRNA-seq) technology will enable high-throughput transcriptome profiling of the *Pax7*^+^ progenitors as well as their descendants, and at the same time allows cell type determination. In this work, we labeled Pax7-expressing cells at E9.5 using the *Pax7*^*CreER*^*:R26R-stop-EYFP* mice and performed scRNA-seq experiments with sorted YFP^+^ descendent cells at E12.5, E14.5, and E16.5. Our data identified the three cell types previously found to arise from somitic mesodermal Pax7-expressing cells, and their lineage progression trajectories were reconstructed from a common progenitor. Using the scRNA-seq data, we discovered unique cell surface markers that enabled isolation of early lineage-specified progenitor cells, which subsequently facilitated our functional assays in vitro. Importantly, we generated a roadmap of Pax7 lineage development at single-cell resolution, which expands our understanding of important transcriptional programs and signaling pathways that drive cell fate decisions at an early stage.

## Results

### Pax7 lineage tracing at single-cell resolution captures temporal transcriptomic progression of the cell fate transition from progenitors to three tissue lineages

**Fig. 1:**
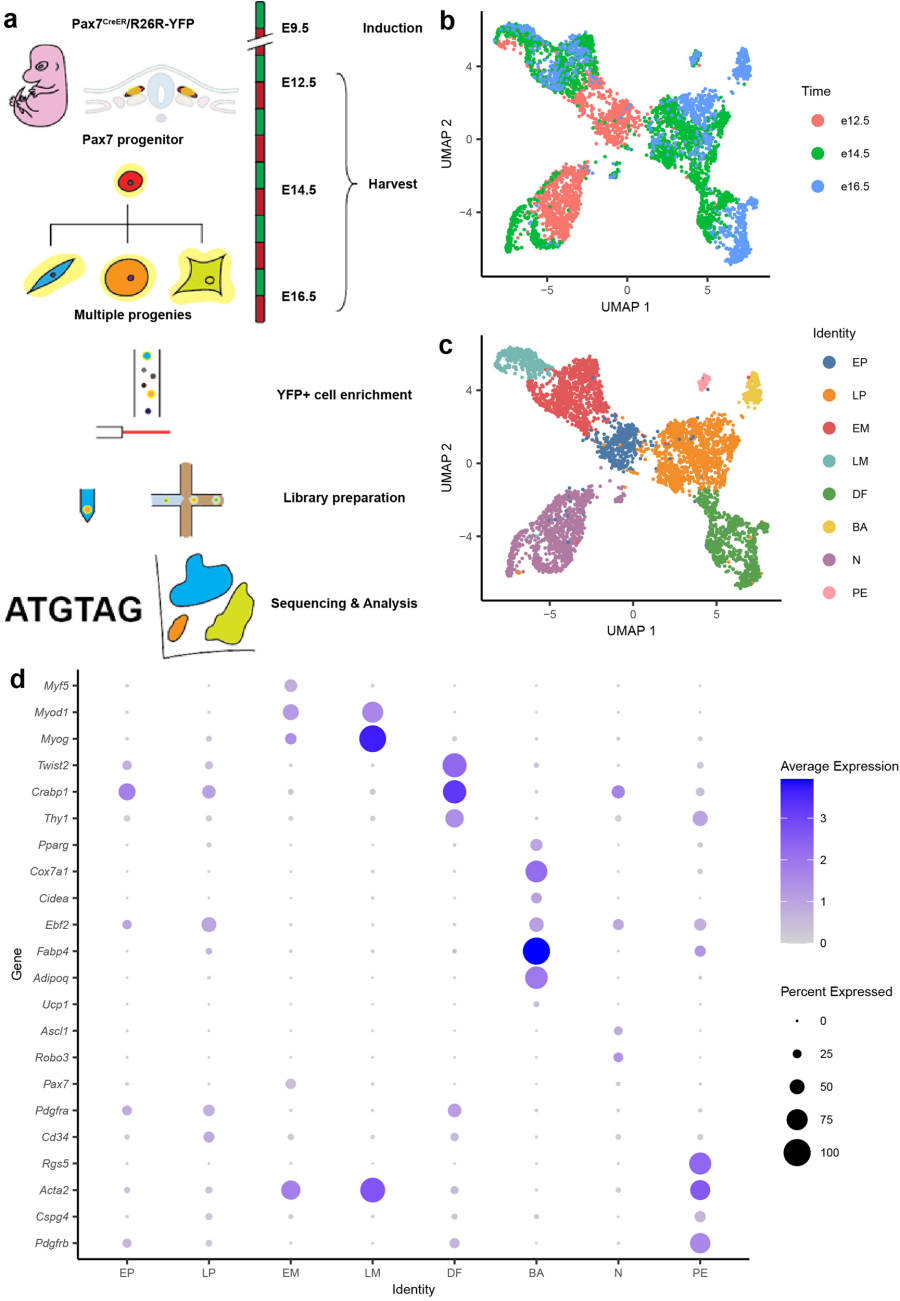
Workflow and cell type identification in Pax7 lineage. **a** Workflow schematic of Pax7 lineage profiling. *Pax7*^*CreER*^*:R26R-stop-EYFP* embryos were induced with tamoxifen on E9.5, and harvested on E12.5, E14.5, and E16.5. Progenies of Pax7^+^ progenitor cells were enriched and sorted by YFP signal. YFP^+^ cells were prepared for sequencing using either Smart-seq2 or 10× protocol, followed by bioinformatic analysis. **b** Uniform manifold approximation and projection (UMAP) of YFP^+^ cells from *Pax7*^*CreER*^*:R26R-stop-EYFP* embryos on E12.5, E14.5, and E16.5. Two datasets from Smart-seq2 and 10× protocol were integrated using scVI for visualization and analysis. Cells were labeled by harvest time of embryos. **c** UMAP of YFP^+^ cells from *Pax7*^*CreER*^*:R26R-stop-EYFP* embryos on E12.5, E14.5, and E16.5. Cells were clustered by unsupervised clustering and were named by identity based on gene expression profile. EP, early progenitor; LP, late progenitor; EM, early muscle; LM, late muscle; BA, brown adipocyte; DF, dermal fibroblast; N, neuron; PE, Pericyte. **d** Dot plot of gene expressions that were used to identify clusters. Each row corresponds to the marker genes for different cell types.

The *Pax7*-expressing progenitors from the somitic dermomyotome have been reported to contribute to skeletal muscle, brown adipose tissue, and dorsal dermis^8,11,18^. Using *Pax7*^*CreER*^*:R26R-stop-EYFP* embryos, wherein tamoxifen-induced YFP expression labels progenies of *Pax7*^+^ progenitors (even if *Pax7* itself is no longer expressed), we performed scRNA-seq profiling of *Pax7* lineage progenies at E12.5, E14.5, and E16.5 after induction of YFP at E9.5. To balance the number of cells analyzed while also being able to detect gene expression with high sensitivity, cells harvested at the same developmental time points were profiled using two different technology platforms: Smart-seq2 (ss2) to capture full-length transcripts at high sensitivity^19^, and high-throughput 10× Chromium Single Cell Gene Expression (10×). The overall experimental schematic is shown in Fig. 1a. After integrating the ss2 and 10× data, datasets were clustered to identify subpopulation and visualized using UMAP (Fig. 1b, c); the integrated result shows consistent mixing between datasets, indicating reproducibility between platform technologies (Supplementary Fig. S1a). Based on marker genes expressed by each cluster, we identified cell types of the myogenic (*Myod1* and *Myog* in the Early Muscle (EM) and Late Muscle (LM) clusters), adipogenic (*Fabp4* and *Pparg* in the Brown Adipocyte (BA) cluster) and dermal (*Twist2* and *Crabp1* in the Dermal Fibroblast (DF) cluster) lineages (Supplementary Table S1). In addition, neuronal (*Ascl1* and *Robo3* in the Neuronal (N) cluster) lineage was also identified (Fig. 1d). Cluster identities were later confirmed by in vitro assays (Supplementary Fig. S5b). Together, these approaches confirm that we captured all known major cell types derived from *Pax7*-expressing progenitors in our data.

For cluster annotation, we first examined the expression of four known myogenic regulatory factors (MRF) in the myogenic lineage: *MyoD*, *Myf5*, *Myf6* (MRF4), and *Myog*^20^. Gene expressions of *Myod1*, *Myf5*, and *Myog* were observed as expected with strong expression in EM and LM; *Myf6* expression was absent, as its expression is suppressed around E11.5 and re-expressed at the start of secondary myogenesis^21^. Among them, *Myf5* and *Myod1* are transcription factors that determine the myogenic cell fate in early muscle precursor cells^22^. *Myog*, on the other hand, is expressed upon the onset of myogenic differentiation and marks the late-stage myogenic cells. Only clusters EM and LM, spanning from E12.5 to E16.5, express all three transcription factors. EM expresses both *Myf5* and *Myod1* while LM downregulates *Myf5* and starts to express *Myog* (Fig. 1d; Supplementary Fig. S1b). This observed timing of the regulation of *Myf5*, *Myod1*, and *Myog* expression is consistent with the classical understanding of myogenic lineage commitment and differentiation^23,24^.

*Twist2* is a transcription factor previously reported as a marker for dermal differentiation^25–27^. Its importance in dermis development has been demonstrated in *Twist2*^−/−^ mice with atrophic dermis^28^. Cluster DF expresses high levels of *Twist2* and includes cells from E14.5 and E16.5, indicating its dermal identity (Fig. 1d). Like *Twist2*, retinoic acid-binding protein *Crabp1* (Fig. 1d) is another known gene marker for the dermal cell population^25^, and it is also expressed by cluster DF, although its role in dermis development remains unclear^25^. Furthermore, Thy1 (Cd90) has previously been described as a surface marker of dermal fibroblasts^29^, and was also found to be enriched in DF (Fig. 1d). The emergence of dermal fibroblasts from somitic mesoderm Pax7^+^ progenitors has not been molecularly characterized before. All evidence strongly suggests that DFs are indeed dermal fibroblasts.

*Pax7*^+^ precursors are also known to give rise to BAT, but not WAT^8,11^. To distinguish between the two, we first examined the expression of *Ucp1*, which encodes a protein that uncouples mitochondria inner membrane potential and generates heat in the BAT^8,30,31^. We only found a small number of cells expressing *Ucp1* in the adipogenic cell clusters BA (Fig. 1d); in line with our observations, a previous report also showed low *Ucp1* mRNA level in E16.5 embryonic BAT, only to be significantly higher from E17.5 onwards^32^. Therefore, we also used a combination of other reported BAT markers, including *Pparg*, *Prdm16*, *Cox7a1, Cidea,* and *Ebf2* to define BAT^8^, and indeed these markers were upregulated in cluster BA (Fig. 1d; Supplementary Fig. S1d). Adipocyte-specific genes such as *Fabp4*, *Adipoq*, and *Plin1*^8,33^ (Fig. 1d) were upregulated as well. Among these, *Ebf2* has been reported as a key lineage-determining factor for BAT and is responsible for establishing the thermogenic gene program^34–36^. Consistent with previous literature showing that its functions are not limited to brown adipocyte precursors^37^, *Ebf2* was expressed not only in BA, but also in the Early Progenitor (EP), Late Progenitor (LP), and N clusters (Fig. 1d).

In addition to the previously reported lineages derived from *Pax7*^+^ precursors, we also observed additional cell populations in our dataset that mostly originate from the earlier developmental time points (E12.5 and E14.5). Furthermore, they express multiple lineage-determining factors but at low levels (Fig. 1d). Their developmental timing and apparent multi-lineage gene expression suggest that these cells are transient developmental progenitor populations that are difficult to be captured without using single-cell technology. Thus, careful examination of these populations may allow us to identify markers for enrichment, and to understand the cellular and molecular regulations of lineage decision. The EP consisted of cells mostly from E12.5 with high level of *Pdgfra* expression (Fig. 1d), which is a marker for mesenchymal progenitors in the adult stage^38^. On the other hand, expression of *Pax7* and *Myf5* in cluster EP is low (Fig. 1d; Supplementary Fig. S1c); and since cells adopting a cell fate other than muscle quickly downregulate Pax7^39^, this suggests that EP are not restricted to myogenic lineage. Lineage-specific genes Twist2 and Ebf2 that mark dermal fibroblasts and brown adipocytes, respectively, were both expressed in this cluster; yet there are no other genes expressed that could identify these EPs as either of these two cell types. Given the first observation of dermal fibroblasts and brown adipocytes that bear differentiated gene markers were at E14.5 and E16.5, EPs from E12.5 are likely to be a mixture of progenitors that can further develop into the myogenic lineage, as well as either dermal or brown adipogenic lineage at later stage. Interestingly, these adult-mesenchymal-progenitor-like cells have not been described in the embryonic development of the mesodermal *Pax7* lineage. The lack of annotation of this population in the literature could be due to the limitations in conventional techniques to identify and isolate these transient state progenitors during development.

In addition to the EP population, LP also did not have a gene expression signature that fit any known terminally differentiated lineages (Fig. 1d). Like the EP described above, the LPs also express the mesenchymal progenitor marker gene *Pdgfra* (Fig. 1d). They are also marked by *Cd34* (Fig. 1d), a common marker for a diverse group of progenitors^40^, which further implies their transient progenitor identity. Interestingly, these cells emerge at later developmental time points (E14.5 and E16.5) in our data (Fig. 1b, c). Similar to EP, they express lineage-specific transcription factors *Ebf2* and *Twist2* (Fig. 1d), which marks BAT and dermal lineages respectively, but each lineage-specific factor is expressed higher and in more cells than the EP, while also not expressing Pax7. We reasoned that these LP cells may be later stage bipotent progenitors at the diverging point of lineage progression towards either dermal or brown adipogenic lineage, but not myogenic lineage anymore. Further validations were performed to demonstrate this in the later part of this study.

A population of cells from the developing neural tube and neural crest expresses *Pax7* as well^10,41–43^, and this population of *Pax7*^+^ progenitors in the ectoderm is known to give rise to commissural neurons in spinal cord^42^, in which Pax3/7 restricts their ventral neuronal identity^10,43^, and induce neuron differentiation^44^. It has been demonstrated that induction at E9.5 or later should predominantly label Pax7 progenitors from the somitic mesoderm, and very few from the neural crest^11^. We found cluster N expressing pro-neural gene *Ascl1*^45,46^ and axon guidance receptor gene *Robo3*^47–50^, suggesting that these could be the residual neural crest Pax7-descendants. We later found corresponding cells in the neural tube of the embryo, explaining why neuronal cells were captured even though we removed the head of embryos prior to YFP sorting (Supplementary Fig. S2a). In addition, a small population of cells from E14.5 and E16.5 were found to express *Rgs5*, *Acta2*, and *Pdgfrb*, which are markers of dermal pericytes (cluster PE). Another classical pericyte marker *Cspg4* (*Ng2*) was also expressed at low levels in this population^51^. The origin of these cells is uncertain: on the one hand, dermal pericytes from mesodermal Pax7-expressing progenitor has not been described previously; on the other hand, although pericytes do arise from the neuroectoderm, these pericytes reside in face and forebrain^52^, and since the embryo head was removed prior to sorting, pericytes of neuroectodermal origin should also have been removed. To focus our study on the Pax7 lineage from the somitic mesoderm, we excluded clusters N and PE from further analysis.

### Pseudotime analysis reveals factors involved in temporal cell fate divergence

**Fig. 2:**
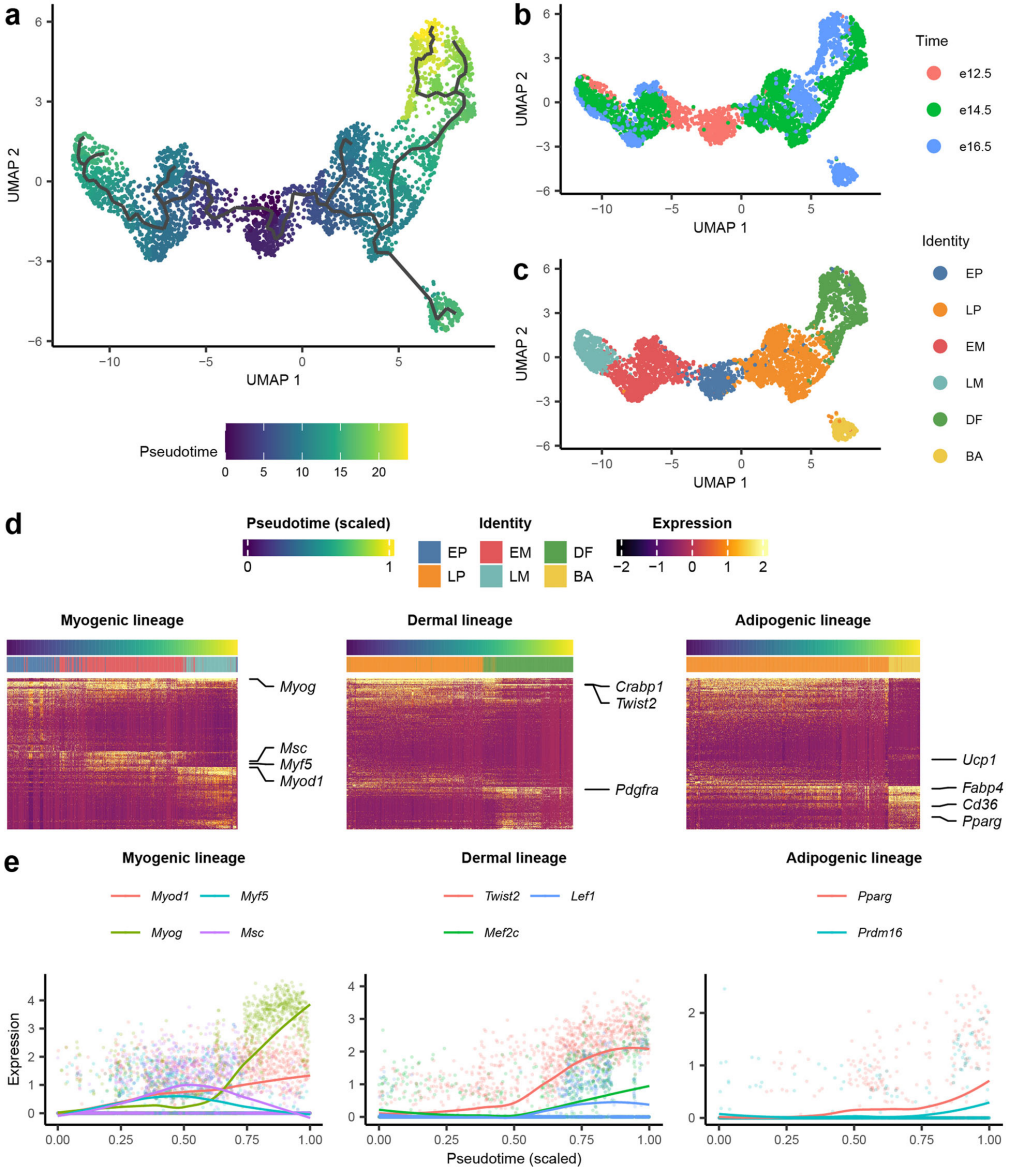
Pseudotime trajectory and its correlation with gene expression in lineages. **a** UMAP of YFP^+^ cells (excluding neurons and pericytes) labeled by pseudotime value with trajectory from Monocle 3. **b** UMAP of YFP^+^ cells (excluding neurons and pericyte) labeled by embryo harvest time. **c** UMAP of YFP^+^ cells (excluding neurons and pericyte) labeled by identity. **d** Z-score heatmap of gene expression in different branches, where rows are genes and columns are cells ranked by pseudotime value. Genes were first fit with generalized additive model (GAM) with ranked pseudotime as independent variable. Genes with the most significant time-dependent model fit (lowest *P*-value) were extracted and clustered by hierarchical clustering. Cells were ordered according to scaled pseudotime value from 0 to 1. Left, Myogenic lineage from EP to EM and LM; Middle, Dermal lineage from LP to BA; Right, Brown adipogenic lineage from LP to DF. **e** Scatter plot of temporally expressed genes obtained from GAM to scaled pseudotime. LOESS line of expression level was shown for each gene.

Single-cell pseudotime analysis examines the transcriptomic differences between each single cell and uses these observed differences to infer gradual transcriptional changes over time. This type of analysis is useful, especially for single-cell studies that focus on temporal molecular dynamics, including developmental processes. We performed pseudotime analysis using the well-established Monocle 3 package^53–55^ and generated an inferred Pax7^+^ cell lineage progression trajectory on the UMAP embeddings from previous analysis (Fig. 2a). Clusters N and PE are likely from a different lineage and outside the scope of our study, and were excluded from analysis. Accordingly, the pseudotime trajectory constructed by the Monocle package matched well with ground truth developmental time points at which the samples were collected (Fig. 2b). Based on this trajectory analysis, E12.5 marks the first branching point of the Pax7 lineage, giving rise to myoblasts in EM marked by *Myf5* and *Myod1* expression in one branch and the *Pdgfra*-expressing EP in the other. No DF or BA cells are present at this stage. Subsequently, E14.5 marks another branching point, where DF first emerge, and we speculate that they originate from the *Pdgfra*-expressing progenitors (LP). E16.5 marks the final branching point, and at this time point we identified BA marked by unique, albeit low, and *Ucp1* expression. LP cells persist abundantly from E14.5 to E16.5, and these *Pdgfra*-expressing progenitors appear to give rise to both dermal fibroblasts and brown adipocytes. In summary, the myogenic lineage undergoes commitment the earliest, at E12.5, while dermal and adipogenic lineages appear at E14.5 and E16.5, respectively.

We then extracted temporally expressed genes with pseudotime in each branch (Fig. 2d, e) using GAM (Supplementary Table S2); this indicates that these genes’ expressions change along with the developmental process, and could also include genes that drive or determine important points in the process. It is also well known that cell fate is often determined and maintained by transcriptional networks governed by master transcription factors (TFs). Thus, to better understand the molecular regulation of lineage progression, we selected only the TFs from the temporally expressed gene list for further analysis (Fig. 2e). In the myogenic lineage (Fig. 2e, left panel), we observed two different trends of TF expression: *Msc* (MyoR) and *Myf5* were upregulated at the early stage in EM but downregulated at the late stage in LM. These TFs are known to be specific to early myogenic progenitors and important for muscle lineage specification^56^, instead of myotube formation. Although only the expression pattern of *Msc* but not its role in dermomyotome myogenesis was previously described^57^, it was shown to coordinate the expression of *Myf5* and *MyoD* during mouse craniofacial development^58^. *Myod1*, *Myog*, and *Mef2c* were found to be temporally expressed at the late stage in LM; although *E2f8*, *Sox6*, and *Sox8* were not found in the gene list, they were nonetheless upregulated at the same time (Fig. 2e; Supplementary Fig. S2b). Among these TFs, *Myod1*, *Myog,* and *Mef2c* are known myogenic regulators that contribute to myogenic differentiation and muscle formation^20,21,59^, while *Sox6* is crucial for muscle fiber type differentiation^60^, and *Sox8* regulates embryonic muscle development^61^. In the dermal lineage (Fig. 2e, middle panel), in addition to the known master regulators, *Twist2* and *Lef1*, we found that *Mef2c* is expressed along with pseudotime as well. Although *Mef2c* is a known regulator for myogenesis^59^, its role in dermal fibroblast development is yet to be investigated. In the brown adipogenic lineage (Fig. 2e, right panel), we found that *Pparg* and *Prdm16* were upregulated along the pseudotime (Fig. 2e) as expected. The expression pattern of *Srebf1* (Supplementary Fig. S2b), although not in the temporal expressed gene list in brown adipogenic lineage, is also in agreement with previous finding that it promotes expression of genes related to fatty acid metabolism, as well as augments transcriptional activity of *Pparg*^62^. Despite having low expression, *Ucp1* still showed an upregulated trend vs pseudotime in brown adipogenic lineage.

Gene ontology (GO) and Kyoto Encyclopedia of Genes and Genomes (KEGG) term enrichment analysis of pseudotime-correlated genes along different lineages also revealed distinct signaling pathways and TFs that are likely involved in regulating lineage progression (Supplementary Fig. S2c–e). In the myogenic lineage, the target genes of *Myog*, *Myod1,* and *Myf6* were enriched (Supplementary Fig. S2c). As for the dermal lineage, components of the Wnt signaling pathway were enriched (Supplementary Fig. S2d), which is also consistent with the existing literature on skin and hair follicle development^63^. In the brown adipogenic lineage, genes that play a role in the PPAR signaling pathway and fatty acid beta-oxidation were enriched (Supplementary Fig. S2e), including direct target genes of *Pparg*.

### Transition between early and late progenitors from E12.5 and E14.5 is gradual and indicates primarily extracellular matrix reorganization activity

**Fig. 3:**
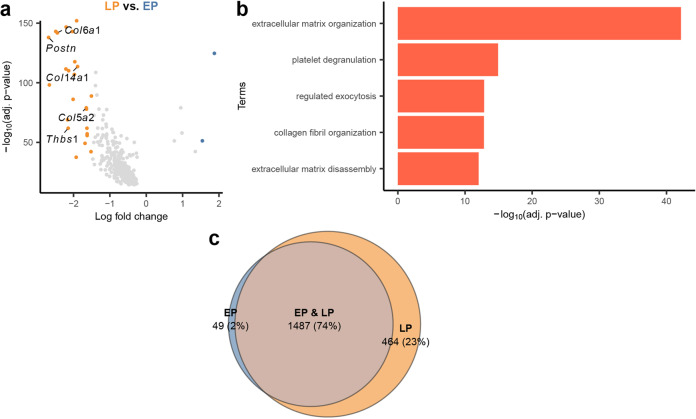
Differential expression analysis and enriched gene ontology between LP and EP. **a** Volcano plot of DEGs between LP and EP. ECM-related genes such as thrombospondin, periostin, and collagens are shown on plot. Genes with adjusted *P*-value < 0.1 were colored by orange (log-fold change > 1) or blue (log-fold change < –1), whereas ≥ 0.1 were colored as gray. **b** Bar plot of gene ontology terms enriched with DE genes of LP (log-fold change > 0). **c** Venn diagram of highly variable gene sets between EP and LP. Highly variable genes between two populations were selected. Genes with raw counts > 3 were kept as set in the diagram.

Since EP and LP arise at different developmental time points, looking for differentially expressed genes between them can help us understand the lineage progression in these progenitors. By performing differential gene expression analysis between EP and LP (Supplementary Table S1), we found upregulation of extracellular matrix (ECM)-related genes in LP, e.g., thrombospondin (*Thbs1*), periostin (*Postn*), and collagen genes (Fig. 3a). GO enrichment analysis of the upregulated genes in LP also showed collagen fibril organization and extracellular matrix organization programs (Fig. 3b; Supplementary Table S3). The importance of the matrix environment in differentiation of pluripotent and multipotent cells was previously investigated, and it has been shown that the elasticity of the ECM greatly affects cell fate^64^. In muscle, ECM plays an important role in muscle differentiation, while the ECM composition in both epidermis and dermis can control epidermal stem cell fate by regulating stem cell anchorage. Deletion of ECM-related proteins could result in skin atrophy and reduced keratinocyte proliferation^65,66^. ECM in the microenvironment can also drive adult human bone marrow-derived MSCs to brown adipocytes^67^. Even though most previous research did not particularly investigate the function of ECM in the cell fate determination of the three lineages described herein, our findings at least support the idea that LP are progenitor cells preparing for lineage differentiation by undergoing ECM reorganization. Interestingly, differential analysis between LP and EP was one-sided towards LP expressing more genes differentially. By examining the highly variable genes in EP and LP, we discovered that 74% of highly variable genes are shared between EP and LP, and while LP has 23% genes uniquely expressed, only 2% of genes are uniquely expressed by EP — in other words, 98% of the genes expressed by EP cells are also expressed by LP cells (Fig. 3c). As a comparison, the same analysis done on EM and LM, as well as BA and DF yield significantly high percentage of non-overlapping genes between each group, even for EM and LM of the same myogenic lineage (Supplementary Fig. S3a, b). This strengthens the idea that the transition from EP to LP is primarily in priming for cell differentiation rather than a distinct change in cell type.

### Combination of surface markers helps enrich different populations including progenitors that can produce different cell-types in vitro

**Fig. 4:**
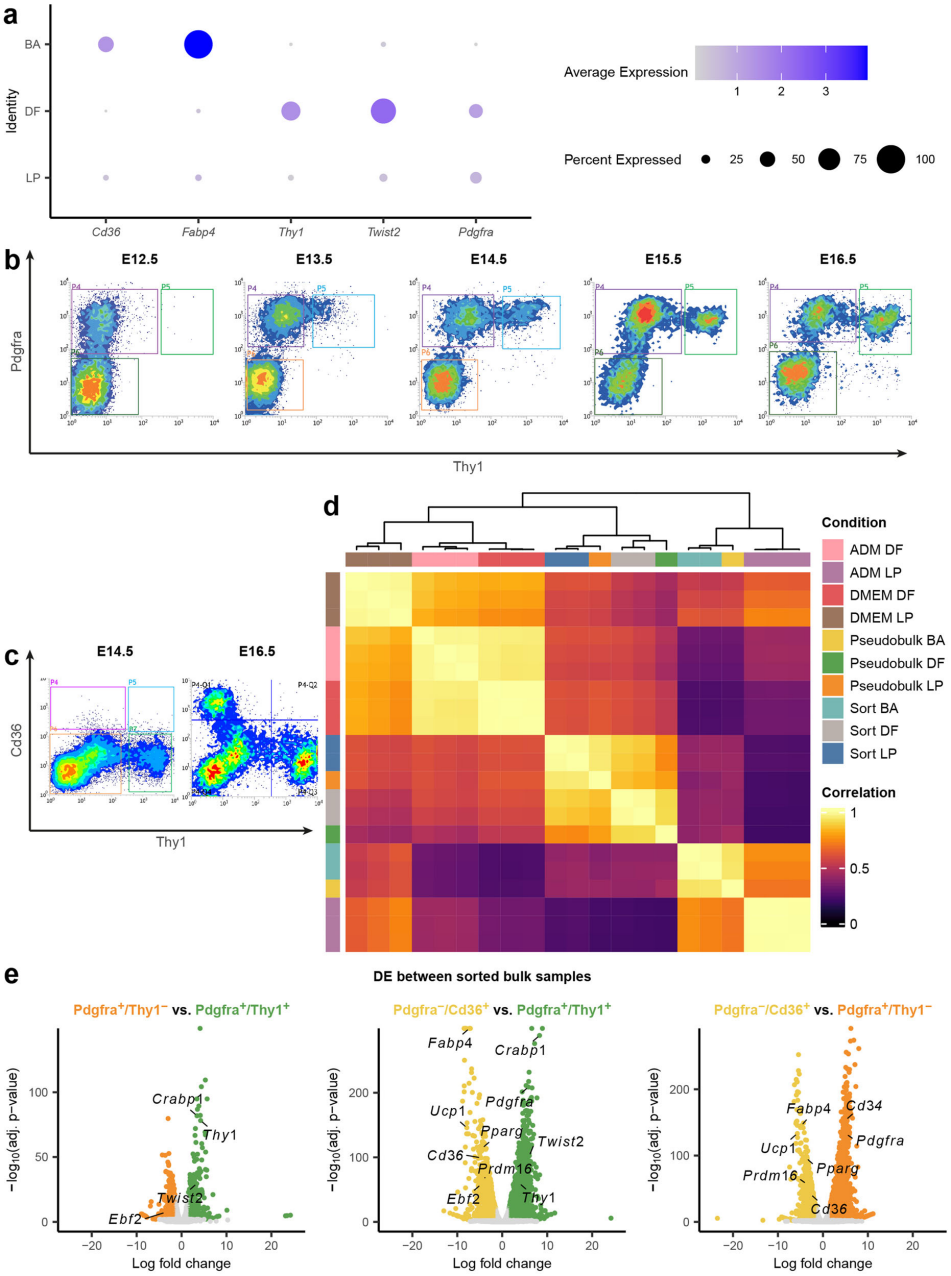
BA and DF surface marker validation. **a** Dot plot showing expression level of *Pdgfra*, *Twist2*, *Thy1*, *Fabp4,* and *Cd36* in LP, DF and BA. **b** FACS plot of YFP^+^ cells from E9.5 Pax7-traced mouse embryos on E12.5, E13.5, E14.5, E15.5, and E16.5. Cells were stained with Thy1 and Pdgfra antibodies to validate the presence of surface markers. *X*-axis indicates the signal from Thy1 antibody while *y*-axis indicates the signal from Pdgfra antibody. **c** FACS plot of YFP^+^ cells from E14.5 and E16.5 that were stained with Thy1 and Cd36 antibodies. *X*-axis indicates signal from Thy1 antibody while *y-*axis indicates signal from Cd36 antibody. **d** Correlation plot of bulk and pseudobulk samples. After FACS sorting with the following surface marker combination: Pdgfra^+^/Thy1^+^, Pdgfra^+^/Thy1^−^, and Pdgfra^−^/Cd36^+^, samples were either prepared for sequencing immediately or cultured. Each row represents one bulk sample. Rows annotated with the same color indicate replicates within the same condition. Gene expression was Z-score normalized. **e** Volcano plots of differentially expressed genes between sorted samples. Genes with adjusted *P*-value < 0.01 were colored by corresponding cluster color (log-fold change > 1.5 or < −1.5), whereas ≥ 0.01 were colored as gray. Lineage markers and surface markers were labeled adjacent to corresponding dots.

Reliable cell surface markers that can be used for sorting and enrichment are essential for performing additional validation and functional characterization of specific cell types. Differentially expressed surface markers found for each cluster could potentially be used to isolate myogenic, dermal, and adipogenic lineages for further downstream analyses. Through our analysis, we identified potential markers for each of these clusters-of-interest, and subsequently used them for FACS sorting: *Thy1* and *Cd36* are preferentially expressed in the DF and BA populations respectively; *Pdgfra* is differentially expressed in LP and DF (Fig. 4a); *Cd34*, a gene that was previously reported to be specifically expressed in multiple progenitor cell types^68^, was found to be differentially expressed in LP (Fig. 1d). Between the dermal and adipogenic lineages, we found that *Thy1* was enriched in DF (Fig. 4a), while *Cd36*, a marker known to be expressed in brown adipocytes responsible for thermogenesis^69^, was enriched in BA (Fig. 4a). Combining these surface markers appropriately allowed us to specifically enrich for each cell type by FACS. Pdgfra^+^/Thy1^+^ selection yields DF, and Pdgfra^−^/Cd36^+^ yields BA. EP and LP are similar to each other in terms of gene expression profiles but emerge at different time points during development. Therefore we isolated the LP cells by sorting Pdgfra^+^/Thy1^−^ specifically at E14.5. Note that at this time point, most but not all cells in the LP cluster express Pdgfra and lack Thy1, and despite the stochastic expression of *Pdgfra* in the LP cluster, this cluster could not be further subclustered into subtypes. While *Pdgfra* RNA is not expressed by all the cells in this cluster, and cell sorting with the Pdgfra^+^/Thy1^−^ combination cannot exhaustively extract all the LP cells, we note that more differentiated cell clusters express both Pdgfra and Thy1, therefore including Thy1^−^ as a sorting gate can achieve enrichment of the LP cells from other cell types for downstream investigation and manipulation. In this case, the timing is particularly important, as Pdgfra^+^/Thy1^−^ cells from a different time point may not be LPs.

As expected, during sorting, Pdgfra^+^ cells were found in embryos from all time points sampled, but only embryos from E13.5 onwards contained cells that were also positive for Thy1 (Fig. 4b). Similarly, Cd36^+^ cells were not found at E14.5; they appear in E16.5 embryos as a distinct population from Thy1^+^ cells (Fig. 4c). Further validation using real-time qPCR (RT-qPCR) on sorted populations showed that *Twist2* and *Ebf2* were upregulated in Thy1^+^ and Thy1^−^ cells respectively, which is also consistent with scRNA-seq results (Supplementary Fig. S5a, b). We also confirmed that these putative multipotent progenitors are distinct from those giving rise to myogenic progenitors by performing sorting with an established marker of skeletal muscle, Itga7^70^ (Supplementary Fig. S4a). Using RT-qPCR, we found that the myogenic marker genes *Myod1* and *Myog* were both highly expressed in Itga7^+^ cells while being downregulated in Pdgfra^+^ cells (Supplementary Fig. S5b). To confirm that our sorting strategy allowed us to enrich for the target LP population, we performed bulk RNA-seq on sorted E14.5 LP (Pdgfra^+^/Thy1^−^), E16.5 DF (Pdgfra^+^/Thy1^+^), and E16.5 BA (Pdgfra^−^/Cd36^+^) cells. The transcriptomes of the bulk samples that were purified by FACS highly correlated with that of pseudobulk transcriptomes generated from corresponding cell clusters in the single-cell data (Fig. 4d; Supplementary Table S4). The expression patterns of marker genes for LP, DF, and BA were consistent between datasets generated by scRNA-seq and bulk RNA-seq (Fig. 4d).

**Fig. 5:**
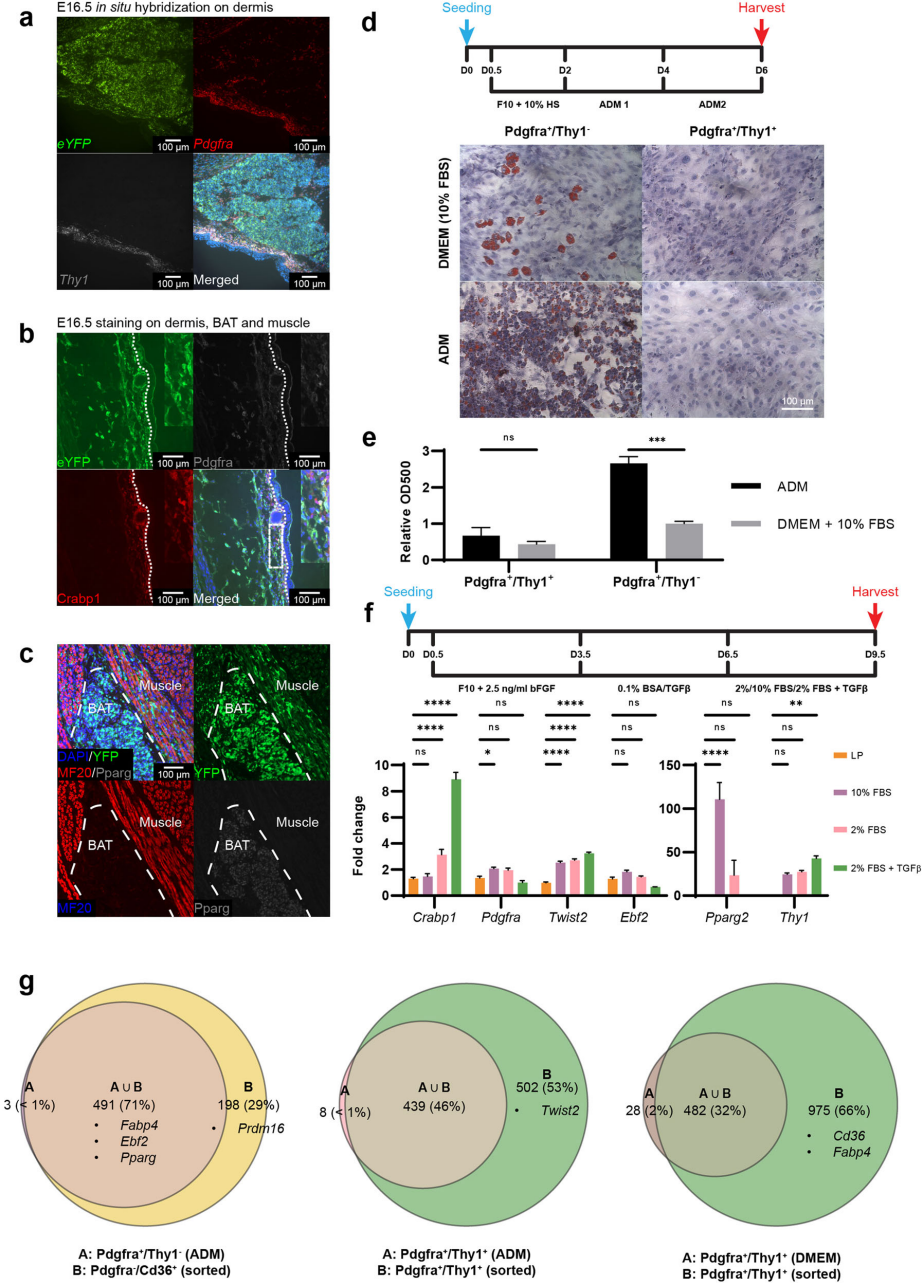
Bulk RNA-seq analysis of cells sorted by surface marker. **a** In situ hybridization of E16.5 mouse embryo showing dermis. *eYFP*, *Pdgfra,* and *Thy1* signals were observed at dermis while only DAPI (blue color) was observed at epidermis. **b** Section staining of E16.5 mouse embryo showing dermis. eYFP, Pdgfra, and Crabp1 signals were observed at dermis while only DAPI (blue color) was observed at epidermis. **c** Section staining of E16.5 mouse embryo showing muscle fiber and brown adipose tissue. While DAPI and YFP signal were observed at both locations, muscle and brown adipose tissue were respectively stained by MF20 and Pparg antibodies. **d** In vitro culture assay of E14.5 YFP^+^ cells sorted by the combination of Pdgfra and Thy1 antibodies. Scale bar, 100 µm. **e** Bar plot of Oil Red O intensity between cultured samples. Student’s *t*-test was performed between two culture conditions. Values are means ± SEM, *n* = 3; ns not significant, ***P < 0.001. **f** Culture schematic and RT-qPCR result of E14.5 Pdgfra^+^/Thy1^−^ cells (LP) before and after TGFβ1 ligand treatment. Two-way ANOVA was performed followed by Dunnett’s multiple comparisons test. Values are means ± SEM, *n* = 3; ns, not significant; **P < 0.01,***P < 0.001, ****P < 0.0001. **g** Venn diagrams of highly variable gene sets between sorted and cultured samples. Highly variable genes between two populations were selected. Key genes found in the corresponding sets are listed. Genes with normalized expression data > 0.3 were kept as set in the diagram.

In situ hybridization and immunostaining of E14.5 and E16.5 embryo sections validated the expression of YFP, Pdgfra, Crabp1, and Thy1 mRNA/protein specifically in dermis but not epidermis (Fig. 5a, b; Supplementary Fig. S5d), as well as the presence of YFP^+^ cells at BAT and muscle region, in which Pparg and MF20 were expressed respectively (Fig. 5c). Most importantly, LP cells (Pdgfra^+^/Thy^−^) sorted from E14.5 had the potential to form the brown adipogenic lineage since Oil Red O oil droplets were readily formed when such cells were cultured in the adipogenic medium (ADM) (Fig. 5d, e). In comparison, the terminally differentiated DF cells (Pdgfra^+^/Thy1^+^) cells lost this adipogenic potential (Fig. 5d, e). Interestingly, the LPs could also give rise to lipid droplet containing cells when cultured in DMEM, albeit the proportion of lipid-containing cells generated was much lower (Fig. 5d, e). We also tested culturing of sorted LP (Pdgfra^+^/Thy1^−^) cells in media with TGFβ1 supplement^71^ to assess their potential for fibrogenic differentiation, and indeed, expression of dermal fibroblast markers such as *Crabp1* and *Twist2* increased in this culture condition while *Pparg2* decreased (Fig. 5f). This suggests that environmental cues and external regulatory factors play important roles in adipogenic or fibrogenic fate commitment in vitro. Pdgfra^+^/Thy1^+^ and Pdgfra^+^/Thy1^−^ cells cultured in both ADM and DMEM in vitro were also sequenced in bulk to inspect the effect of environmental cues on the transcriptomic profile of the late progenitors and dermal fibroblasts. The transcriptomic profiles of cells cultured in vitro in ADM and DMEM were less correlated with that of freshly sorted cells or the pseudobulk generated from scRNA-seq (Fig. 4c), suggesting the in vitro culture conditions cannot fully mimic the signaling cues in vivo.

### Differentiated cell types derived from sorted progenitors cultured in vitro share similar transcription profiles with their in vivo counterparts

To further investigate whether LPs or DFs are undifferentiated cells or cells with committed lineage, DE analysis was performed between sorted cells and cultured cells (Supplementary Fig. S5c and Table S5) and were found to share highly variable genes (Fig. 5g). LP (Pdgfra^+^/Thy1^−^) cells cultured in ADM differentially expressed *Ucp1*, *Fabp4*, and *Cd36,* whereas *Ebf2* and *Prdm16* were differentially expressed in freshly sorted BA (Pdgfra^−^/Cd36^+^) cells. Given that *Ucp1* is expressed in mature BAT^8^, in vitro culture of Pdgfra^+^/Thy1^−^ cells showed LP cells were induced towards adipogenesis further than freshly sorted BA (Fig. 5g). To see if ADM could induce adipogenesis even in a committed lineage, differential expression analysis was performed between freshly sorted Pdgfra^+^/Thy1^+^ (DF) cells and cultured DF cells, both in ADM and DMEM (Supplementary Fig. S5c and Table S5). Although *Fabp4*, *Adipoq,* and *Cd36* were differentially expressed in ADM cultured cells, *Ucp1*, *Cox7a1,* and *Prdm16* was not differentially expressed. This shows that ADM induction in cultured DF had a pro-adipogenic effect but could not completely drive cells toward BAT as it could in cultured LP. The key markers and gene expression features of these in vitro derived DF and BA cells resemble that of their in vivo counterparts, and important phenotypic features such as the formation of lipid-containing droplets were also recapitulated in vitro for the cultured BA cells. In addition, sorted cells expressed higher number of genes compared to the cultured counterpart (Fig. 5g), suggesting the remaining transcriptomic differences between the cultured and embryo-derived cell populations for both BAs and DFs highlight the critical role of environmental cues in the differentiation and maturation process during development.

### Silencing *Rgcc* inhibits brown adipocyte development from late progenitor

**Fig. 6:**
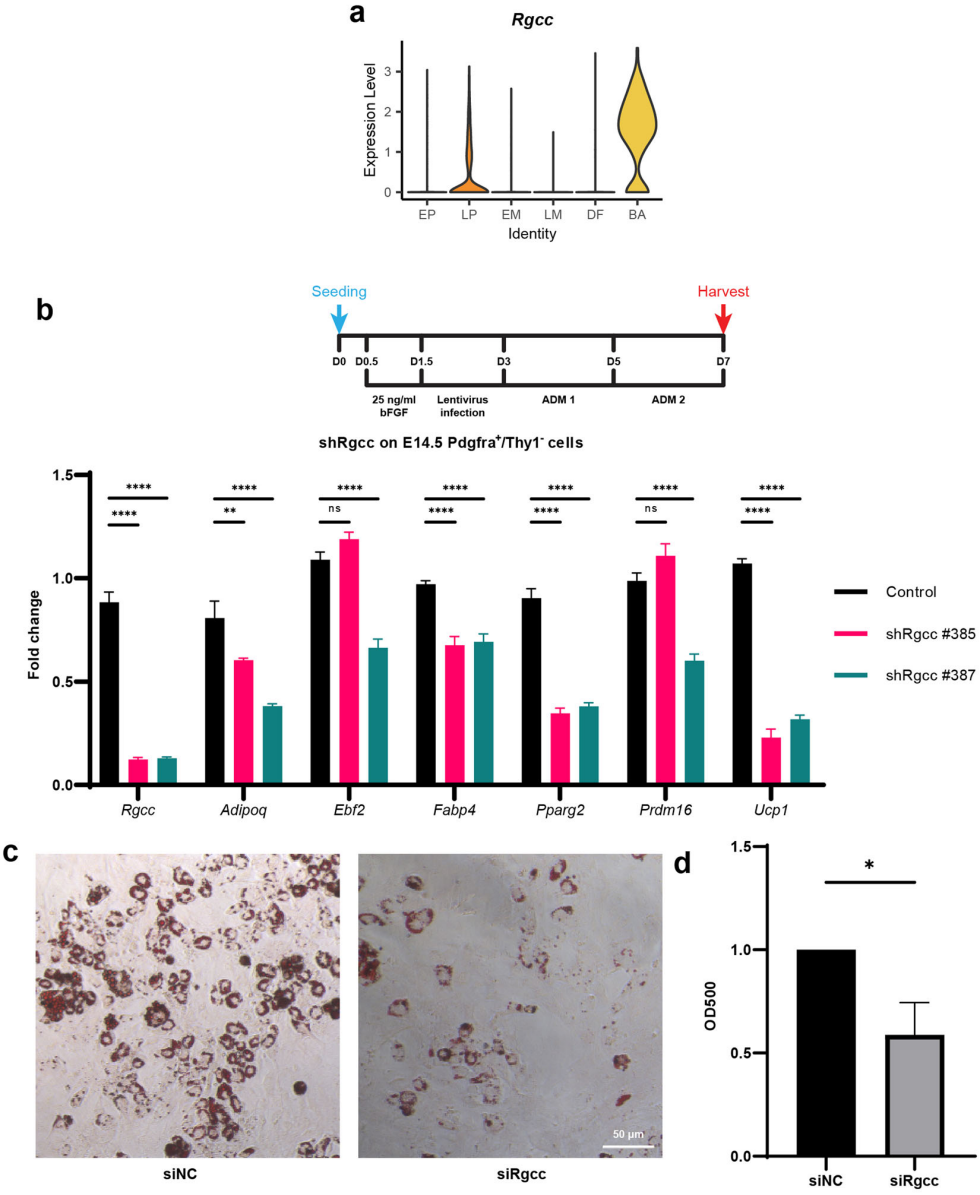
Cell differentiation inhibition by *Rgcc* silencing. **a** Violin plot of *Rgcc* expression in YFP^+^ cells. **b** Culture schematic and expression of *Rgcc* by RT-qPCR on E14.5 Pdgfra^+^/Thy1^−^ cells after shRgcc knockdown by lentivirus. Infected cells were cultured in ADM before harvest. Two-way ANOVA was performed, followed by Dunnett’s multiple comparisons test. Values are means ± SEM, *n* = 3; ns, not significant; **P < 0.01, ***P < 0.001. **c** Oil Red O staining of Pdgfra^+^/Thy1^−^ cells after siNC (control) and siRgcc knockdown. **d** Quantification of Oil Red O staining. Student’s *t*-test was performed between siNC and siRgcc OD500 intensity. *P < 0.05.

We then sought to investigate key regulators of lineage determination in the Pax7^+^ lineage, and we are especially interested in understanding how BA and DF lineages are specified. To do this, we performed differential gene expression analysis between BA and LP and selected some transcription factors and upregulated genes (Supplementary Table S1) for siRNA knockdown experiment and examined the cell fate choice after gene silencing. Among these candidate genes, *Rgcc* expression was unique in BA (Fig. 6a) and a previous study showed significant upregulation of *RGCC* in adipose-derived stromal cells throughout the adipogenic differentiation^72^, suggesting that it could play a regulatory role in the BA differentiation process. As such, we performed shRNA knockdown of *Rgcc* in the Pdgrfa^+^/Thy1^−^ (LP) cells sorted from E14.5 embryos cultured in ADM conditions to maximize the adipogenic differentiation and examined the cell fate after *Rgcc* perturbation (Fig. 6b). With both shRgcc that we designed, adipogenic-related genes, including *Ucp1*, were downregulated. We also performed siRNA knockdown of Rgcc in sorted LP cells, and after 6–8 days of culture we used Oil Red O staining as adipogenic readout (Fig. 6c, d). Consistent with our hypothesis, cells with *Rgcc* knockdown indeed had fewer lipid droplets, suggesting *Rgcc* positively regulates adipogenesis.

### Wnt5a suppresses adipogenesis from late progenitor

**Fig. 7:**
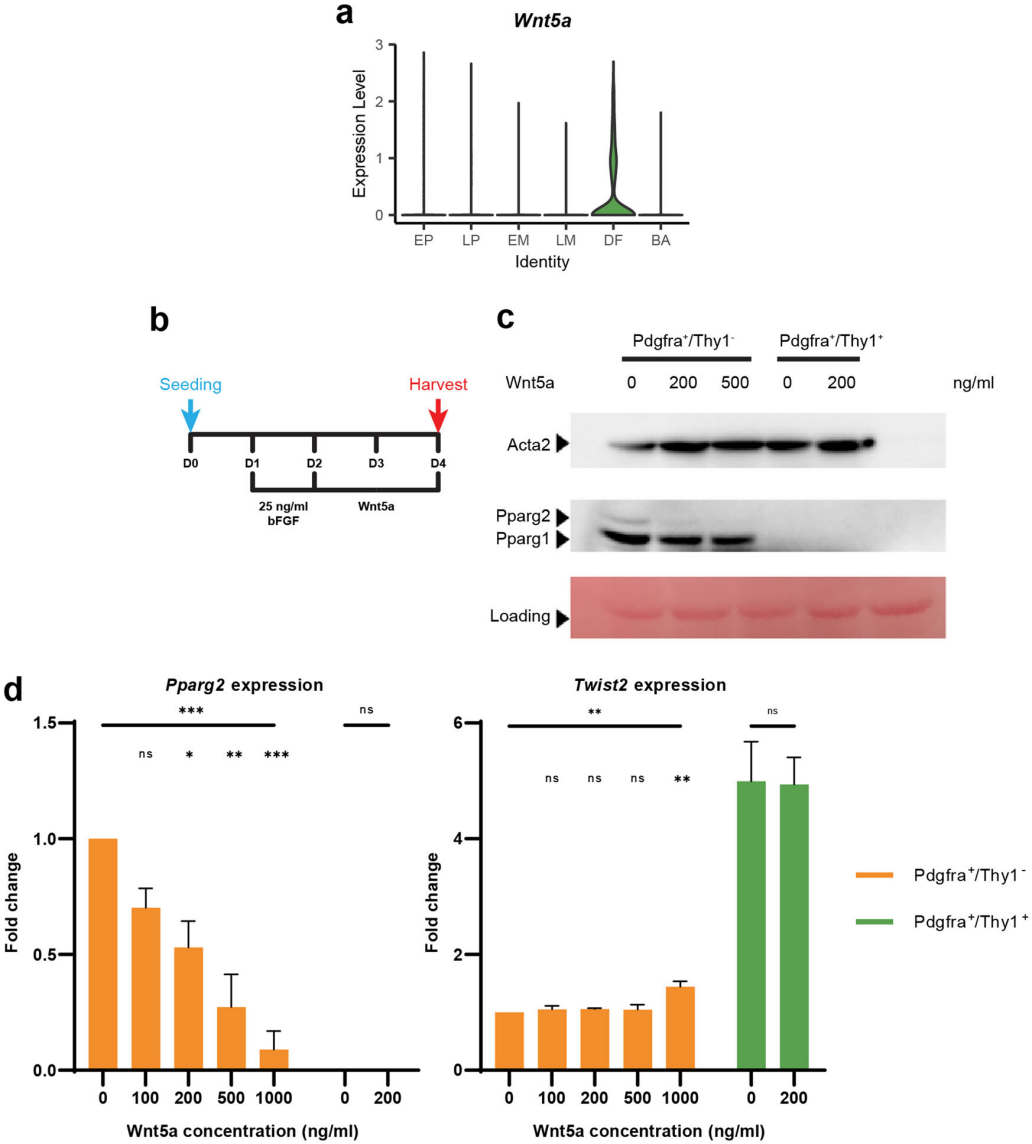
Wnt5a inhibits adipogenic gene expression in embryonic multipotent progenitors. **a** Violin plot of *Wnt5a* expression in Smart-seq2 and 10× datasets. **b** Scheme of Wnt5a treatment on embryonic progenitor cells. E14.5 embryos were harvested, and both Pdgfra^+^/Thy1^−^ and Pdgfra^+^/Thy1^+^ populations were sorted. Cells were treated with 2.5 ng/mL bFGF to reach confluency. Wnt5a was then applied and 48 h after treatment, cells were harvested for analysis. **c** Embryonic cells were harvested and subjected to Western blot assay. Ponceau S staining was used as loading controls. **d** RT-qPCR analysis of Wnt5a-treated embryonic progenitor cells in **b**. One-way ANOVA was performed on Pdgfra^+^/Thy1^−^ cultured cells, followed by Dunnett’s multiple comparisons test. Student’s *t*-test was performed in Pdgfra^+^/Thy1^+^ cultured cells. Values are means ± SEM, *n* = 3; ns, not significant; **P < 0.01, ***P < 0.001.

We also examined the key signaling events that could potentially regulate lineage specification. Among all the major developmental signaling pathways we investigated, the Wnt signaling pathway was found to be enriched in DF (Supplementary Fig. S2d). Therefore, we examined the expression of multiple Wnt ligands in our single-cell data and found Wnt5a was highly expressed in the DF (Fig. 7a). Previous reports have shown inhibition of adipogenesis in mesenchymal precursor cells by Wnt5a secreted from adipose tissue macrophages^73^. To test whether Wnt5a indeed suppresses the adipogenic fate of LPs, we sorted LP cells (Pdgfra^+^/Thy1^−^) at E14.5 and treated the cells with recombinant Wnt5a protein. In parallel, differentiated Pdgfra^+^/Thy1^+^ dermal fibroblasts were also sorted and treated as control. We retrieved the cells 48 h after Wnt5a treatment (Fig. 7b) and subjected them to RT-qPCR analysis. As expected, *Pparg2*, an adipocyte-specific *Pparg* isoform^74–76^, was barely detectable in DF (Pdgfra^+^/Thy1^+^) cells both with and without Wnt5a, consistent with their committed cell fates (Fig. 7d). In the LP (Pdgfra^+^/Thy1^−^), however, there was a dose-dependent inhibition of Pparg2 expression after Wnt5a treatment (Fig. 7d). The inhibition of Pparg2 protein expression by Wnt5a was also confirmed using Western blot assay (Fig. 7c). Wnt5a ligand treatment also upregulated expression of *Twist2* in LP (Fig. 7d). Together, these results indicate the upregulation of Wnt5a in multipotent progenitor cells negatively regulates genes involved in adipogenesis, but whether the cell fate will be directed to dermal fibroblasts still needs further investigation.

## Discussion

Previous Pax7 lineage-tracing studies have highlighted the multipotency of *Pax7*^+^ progenitor cells in dermomyotome that can develop into skeletal muscle, interscapular brown adipose tissue, and dorsal dermis^11,77^. These studies revealed diverse cell fates in the Pax7 lineage during embryonic development, but the precise time points when the progenitor cells commit to a particular lineage are still inconclusive. By tracing and probing the *Pax7* lineage at multiple developmental time points at single-cell resolution, we here showed the transcriptomic profile across *Pax7* lineage development. Referencing previously studied gene markers we identified different cell populations of the myogenic, dermal, and adipogenic lineages, and defined the timing of lineage commitment during embryonic development. Focusing on the three lineages, we found that lineage commitment of *Pax7*^+^ progenitors occurs between E12.5 and E16.5. During this time course, the myogenic lineage branches out at E12.5 with *Myf5* upregulation, while the remaining cells undergo *Pdgfra* upregulation and commit into the dermal lineage at E14.5 with *Twist2* upregulation, or into the brown adipogenic lineage with *Pparg* upregulation at E16.5. Our single-cell analysis agrees with previous findings on the cell fate choices of the *Pax7* lineage during embryonic development. In addition, we reveal for the first time the temporal patterns of the commitment for each lineage.

We noticed the contribution of E12.5 cells to both EM and LM of the myogenic lineage, whereas cells from E14.5 and E16.5 mainly contribute to LM. The pseudotime trajectory analysis suggests that *Myog*^+^ cells (LM cluster) are derived from *Myf5*^+^/*Myod1*^+^ cells (EM cluster), which is consistent with existing knowledge about myogenic lineage progression from proliferating myoblasts to differentiating myocytes or differentiated myotubes. Meanwhile, *Myf5*^+^ cells at E12.5 can continue to differentiate into *Myog*^+^ cells captured at E14.5 and E16.5. Previously, *Myf5*-expressing cells were shown to give rise to both skeletal muscle and brown adipose^9^. Similarly, in the Myf5 lineage, previous studies demonstrated that Ebf2 protein is expressed in E11.5 embryos and that an *Ebf2*^+^ population dramatically expands at E12.5^36^, which implies that the brown adipogenic cell fate is established by E12.5; *Pparg*, a master regulator of adipogenesis, was reported to be expressed in the Myf5 lineage as early as E14^36^. Our study of the Pax7 lineage showed high expression of *Ebf2* in non-neuronal cells at E14.5, suggesting that cell fate commitment likely occurs much later than E12.5 in the Pax7 lineage. *Pparg* was neither detected by real-time PCR, nor by scRNA-seq in cells from E14.5. Instead, significant *Pparg* expression was observed only in cells derived from E16.5, suggesting adipogenic lineage commitment occurs much later than myogenic lineage. This finding suggests that in the Pax7 lineage, active mechanisms exist to inhibit premature differentiation of adipocytes at earlier developmental time points.

Currently, when investigating lineages-of-interest, the lack of surface markers that can be used to prospectively isolate and enrich cells from a given lineage poses critical challenges. Pdgfra is regarded as one of the cell surface markers for brown adipocyte progenitors^78^, yet we here show that in the *Pax7* lineage, *Pdgfra* is in fact expressed in the multipotent progenitors (both EP and LP) at E12.5 and E14.5 and the dermal lineage at E14.5, and that its encoded receptor protein can be used as a cell surface marker to label all of these lineages at specific embryonic time points. Pdgfra, therefore, is not a unique surface marker for the adipogenic lineage arising from *Pax7* progenitors. Furthermore, the gene products of cell surface proteins Thy1 and Cd36 were found to be uniquely expressed in dermal fibroblast and brown adipocyte, respectively. Together with Pdgfra, these two markers were demonstrated to be useful for prospective isolation of dermal fibroblast, brown adipocyte, and their progenitor subpopulations in the Pax7 lineage by FACS. We further performed bulk RNA sequencing and in vitro cell culture experiments on both sorted and cultured cells. Collectively these findings show that using the surface markers we have identified, it is possible to isolate specific lineages of interest; this is especially important for the study of dermal and adipogenic lineages that were previously challenging to isolate due to lack of suitable surface markers.

We also further investigated the lineage potential of the late progenitor and uncovered a novel mechanism that regulates the generation of adipocytes during the development of this lineage. We observed that *Rgcc*, a gene that has been linked to adipogenesis in other contexts, was upregulated in the brown adipocytes. *Rgcc* was previously found to be upregulated during adipogenic induction of adipose-derived stromal cells^72^, as well as the sheep perirenal adipose tissue for both brown and white adipocytes at the beginning and the end of its transformation^79^. Experiments using shRNA and siRNA knockdown of *Rgcc* in late progenitor cells showed that despite not being a transcription factor, gene silencing of *Rgcc* reduced the efficiency of deriving adipocytes from late progenitors in vitro, and reduced expression of *Pparg2* in LP cells (Fig. 6b), supporting the role of Rgcc in adipocyte fate determination in the embryonic Pax7 lineage.

We also found that Wnt5a expression is induced after the branching of dermal fibroblasts. The inhibitory effects of Wnt signaling on adipogenesis are well documented in literature: suppression of the Wnt pathway serves as a prerequisite for proper adipogenic differentiation, and Wnt ligands including Wnt5a have been shown to repress adipogenesis and maintain the pre-adipocyte in an undifferentiated state by downregulating the expression of *Pparg* and *Cebpa*^80–83^. However, the exact Wnt ligands that modulate the fate specification of the *Pax7* lineage progenitors were not known. Treatment of the multipotent LP (Pdgfra^+^/Thy1^−^) cells with Wnt5a ligand strongly inhibited *Pparg2* expression in culture. These results indicate Wnt5a could serve as an important fate regulator to negatively regulate the adipogenic program, thus directing cells to a fibroblast fate. Wnt5a treatment also upregulated *Twist2* expression, though with less dramatic magnitude. This suggests Wnt5a influences cell fate choice mainly by suppression of the adipogenic program, while other extracellular cues may be needed for *Twist2* upregulation. These findings illustrate the feasibility of using scRNA-seq analysis for identifying mechanistically important in vivo factors that play a role in cell fate determination, and they serve as a starting point for further investigation of such mechanisms in developmental.

Fibro/adipogenic progenitors (FAPs), bipotent cells capable of giving rise to fibroblasts and adipocytes, are usually studied in the context of muscle fiber regeneration in the adult muscle tissue^38,84,85^. Prior investigations have demonstrated their ability to adopt either fibrotic or adipogenic fate depending on the environmental cues and that they can contribute to muscle fibrosis and fat accumulation^85^. Strikingly, our data have shown that progenies of *Pax7*-expressing cells that do not commit early into the myogenic lineage can later develop into bipotent progenitor cells that, like FAPs, can give rise to both dermal fibroblasts and brown adipocytes, but in the embryonic tissue. This embryonic FAP-like (eFAP) population has not been identified or characterized previously within Pax7 lineage; using scRNA-seq, we were able to identify surface markers (Pdgfra^+^/Thy1^−^) to isolate them by FACS for in vitro culture and experimental characterization. Based on our subsequent in vitro studies, an increased number of lipid-storing cells being derived from our LP (Pdgfra^+^/Thy1^−^) cell culture in adipogenic medium suggests that the lineage determination of this bipotent eFAP is at least partially driven by the growth environment. Most of the reported investigations of FAPs have focused on cells from hindlimb of adult mice and have found Sca-1 and Cd34 to be surface markers specific for FAPs from this adult tissue type. In our eFAP cell population, only *Cd34* mRNA was expressed while *Sca-1* mRNA was not expressed (Supplementary Fig. S6); existing literature also shows absence of Sca-1 expression in the upper dermis of mice from E12.5 to P2 while Cd34 expression can be seen from E12.5 onwards^86^. This indicates that the embryonic tissue/cells can be significantly different from the adult ones even if they have similar fate potency, and that markers previously found for cell-types-of-interest in adult tissues may not be applicable when studying even the same tissue or cell type in the embryonic context.

In conclusion, we herein present a single-cell transcriptomic analysis on *Pax7* lineages from the developing dermomyotome. Transcriptomic profiling of *Pax7* lineages allowed us to identify cell fate commitment time points for the myogenic, dermal, and adipogenic lineages. We discovered surface markers for robustly isolating distinct cell populations from embryos, thus enabling in vitro culture and functional characterization of these populations. Our analyses also generated candidate genes, including some TFs, that could be involved in determining or reprogramming cell fate. We further demonstrated that *Rgcc* and *Wnt5a* are important for lineage development during embryogenesis. We lastly propose eFAP cells, a previously uncharacterized embryonic bipotent progenitor population marked by Pdgfra^+^/Thy1^−^ expression emerging predominantly at E14.5 that resembles adult FAPs in their lineage potentials. Overall, this work has furthered our understanding of lineage diversification in *Pax7*^+^ progenitor cells during embryonic development and provided new avenues for future in-depth mechanistic studies of cell fate choice during development.

## Materials and methods

### Mouse lines

Pax7^creER^ (Gaka) (stock 017763) and R26-stop-EYFP (stock 006148) mice were from Jackson Laboratory (Bar Harbor, ME, USA). Mice were housed in the Animal and Plant Care Facility (APCF) at Hong Kong University of Science and Technology (HKUST). All the experiments were performed in accordance with protocols approved by the Animal Ethics Committee at the HKUST.

### Lineage tracing

Female and male mice were split 16 h post-mating and designated as embryonic day 0.5 (E0.5). To trace the progeny of Pax7-expressing cells during early developmental stage, a single dose of TMX (75 µg/g body weight) were injected intraperitoneally into the pregnant mice with weight gain > 2 g at E9.5 to initiate Cre-Loxp recombination in Pax7-expressing cells. Embryos were harvested later at E12.5, E14.5, and E16.5.

### Embryonic cell isolation

Tissues previously known for absence of *Pax7* expression such as limbs and viscera were removed. Head of the embryos were also removed. Isolated mouse embryos were minced and digested in sorting medium (Ham’s F10 with 10% horse serum) containing 400 U/mL Collagenase II (Worthington; LS004177) for 1 h at 37 °C in a shaking water bath. For E14.5 or older embryos, additional Dispase (1U/mL; Gibco) was added. The digested embryos were then washed in the sorting medium and filtered through a 40 μm cell strainer (BD Falcon).

### Flow cytometry

Cells in Pax7 lineage were first identified and enriched by YFP signal in FITC channel.

For single-cell RNA-seq, cells were then re-sorted on a BD FACSAria III or Influx^TM^ cell sorter with FITC signal. Multiple embryos were pooled for sorting in both Smart-seq2 and 10× library preparation due to limited YFP^+^ cells per embryo.

### Full-length scRNA-seq library preparation and sequencing

Full-length scRNA-seq libraries were prepared from YFP^+^ single-cells according to Smart-seq2 protocol^87^.YFP^+^ single-cells were sorted into 96-well plate containing 0.2% (v/v) Triton X-100, 10 mM dNTP mix (New England Biolabs), and 10 μM oligo-dT_30_VN. Sorted cells were either proceeded immediately to oligo-dT hybridization and reverse transcription, or snap-freez and stored at −80 °C. After hybridization at 72 °C for 3 min, reverse transcription mix with SuperScript II Reverse Transcriptase (Invitrogen) and template-switching oligo was added immediately to each well followed by reverse transcription. Afterwards cDNA was amplified using 2× KAPA HiFi HotStart ReadyMix (Roche) with 18 cycles. Amplified cDNA were then purified with 0.8× AMPure XP beads (Beckman Coulter) with quality and quantity check on Fragment Analyzer (Agilent) and Qubit Fluorometer (Thermo Fisher Scientific), respectively. Libraries were then completed with Illumina Nextera XT library construction kit (Illumina). Samples were pooled after final PCR amplification and purified with 0.9× AMPure XP beads. The final libraries were sequenced on Nextseq 500 (Illumina) using 75 bp pair-end-reads setting. The sequencing depth of each cell in Smart-seq2 dataset is 1.29 million reads on average.

### 3' scRNA-seq library preparation and sequencing

3' scRNA-seq libraries were prepared from YFP^+^ cells according to the 10× Genomics Single Cell 3' Reagent Kit v2 protocol. Enriched YFP^+^ cells were immediately loaded into Chromium Chip targeting 7000 cells. After cDNA amplification and sample index amplification, libraries were quantified and qualified on Fragment Analyzer (Agilent) and Qubit Fluorometer (Thermo Fisher Scientific), respectively. Libraries were sequenced on Nextseq 500 (Illumina) with the following parameter: 27, 8, 0, 125. The sequencing depth of each cell in 10× dataset I 147k reads on average.

### Bulk RNA-seq library preparation and sequencing

6–8 embryos from the same day were pooled for digestion after removal of limbs, viscera, and head. First enriched by YFP signals, cells belong to Pax7 lineage were further sorted with cell surface markers, including Pdgfra, Thy1, and Cd36. Total RNA of sorted bulk and cultured bulk samples were extracted with NucleoSpin RNA isolation kit (Macherey-Nagel). Sequencing libraries of the bulk samples were prepared in the same way as single-cell Smart-seq2 method; only single-cell input was replaced with total RNA extracted. Similarly, libraries were completed with Illumina Nextera XT library constructed kit (Illumina). The final libraries were sequenced on Nextseq 500 (Illumina) using 75 bp pair-end-reads setting. For cultured group, triplicate wells were used for sequencing, while for E16.5 sorted cells (no culture), technical duplicates were used. Each bulk sample contains an estimate of 20 k cells. Mapped reads were 27 million on average.

### Cell culture

For embryonic cell culture, the 48-well plates were pre-coated with 0.1% gelatin at 37 °C for 1 h. The sorted embryonic cells were cultured in Dulbecco’s modified Eagle’s medium (DMEM) with 10% fetal bovine serum (FBS). The adipogenic differentiation medium (ADM) was described previously^14^. After reaching confluency, the cells were first cultured in ADM I for 2 days and then switched to ADM II. ADM II were refreshed every 2 days.

### Tgfβ1 treatment

LP (Pdgfra^+^/Thy1^−^) cells were cultured in F10 with 10% horse serum supplemented with 2.5 ng/mL bFGF (Prospec) to reach confluency. Cells were then treated with 1 ng/ml Tgfβ1 (Peprotech) in differentiation medium (DMEM with 2% FBS) for 3 days followed by culturing in differentiation medium without Tgfβ1 for 3 days.

### Oil Red O staining and immunostaining

For Oil Red O staining, cells were fixed in 10% formalin, rinsed with ddH_2_O and stained in Oil red O working solution (final 36% in Triethyl phosphate). The cells were then incubated with hematoxylin for 5 min for nuclei staining. For immunostaining, cells were fixed with 4% paraformaldehyde for 5 min, followed by permeabilization in 0.5% PBST and blocking in 4% BSA. Cells were then incubated with primary antibodies overnight at 4 °C. After rinsing with 0.1% PBST for three times, cells were labeled with secondary antibodies for 1 h at room temperature and then subjected to imaging. For tissue section immunostaining, the sample processing procedures are similar to cell immunostaining except 0.3% PBST were used for rinsing. Antibodies used are as follows: APC anti-mouse CD90.2 (Thy-1.2) Antibody (BioLegend, Cat# 140311), PE anti-mouse CD140a Antibody (BioLegend, Cat# 135905), PPAR gamma Monoclonal Antibody (Invitrogen, Cat# MA5-14889), Human/Mouse EBF-2 Antibody (R&D Systems, Cat# AF7006), Biotin Anti-GFP antibody (Abcam, Cat# ab6658), Mouse monoclonal anti-MyoD (Dako, Cat# M3512), Anti-Actin, α-Smooth Muscle antibody, Mouse monoclonal (Sigma-Aldrich, Cat# A5228), PE anti-mouse CD36 Antibody (BioLegend, Cat# 102605), Mouse monoclonal anti-Myh1 (Developmental Studies Hybridoma Bank, RRID AB_2147781), CRABP1 (D7F9T) Rabbit mAb (Cell Signaling Technology, Cat# 13163S), and Mouse PDGF R alpha Antibody (R&D Systems, Cat# AF1062).

### In situ hybridization by RNAscope

RNA-scope experiment was performed following the manual of RNAscope multiplex fluorescent reagent kit V2 assay (Advanced Cell Diagnostics). Briefly, embryo sections were post-fixed with 4% PFA for 15 min, followed by sequential dehydration in 50%, 70%, and 100% ethanol. Sections were then treated with hydrogen peroxide for 10 min at room temperature. Antigen retrieval was performed at 99 °C for 5 min in 1× Target Retrieval Reagent. Sections were then digested with Protease III for 30 min at 40 °C. For probe hybridization, probes (EYFP-C1; Cat# 312131, Mm-Thy1-C2; Cat# 430661-C2 and Mm-Pdgfra-C3; 480661-C3) were diluted according to manufacturer’s instructions and incubated with sections for 120 min at 40 °C. After washing, signal amplification was conducted by sequential hybridization with AMP1 (30 min), AMP2 (30 min), and AMP3 (15 min). For signal development, Opal 520, Opal 620, and Opal 690 (Akoya Biosciences) were diluted at 1:1500 and assigned to C1, C2, and C3, respectively.

### RNA isolation and RT-qPCR

Total RNA for RT-qPCR were extracted from embryonic cells with TRIzol (Thermo Fisher Scientific), followed by cDNA synthesis using ImProm-II reverse transcription system (Promega). Real-time PCR were performed on a Roche LightCycler 480 machine using SYBR green master mix (Roche). Sequence of primers used are as follows: Pparg2 forward: GCATGGTGCCTTCGCTGA; reverse: TGGCATCTCTGTGTCAACCATG. Rarg forward: GGAGCAGGCTTCCCATTCG; reverse: CATGGCTTATAGACCCGAGGA. Twist2 forward: CGCTACAGCAAGAAATCGAGC; reverse: GCTGAGCTTGTCAGAGGGG. Ebf2 forward: GGGATTCAAGATACGCTAGGAAG; reverse: GGAGGTTGCTTTTCAAAATGGG. MyoD forward: CGCTCCAACTGCTCTGATG; reverse: TAGTAGGCGGTGTCGTAGCC. Myog forward: GCAATGCACTGGAGTTCG; reverse: ACGATGGACGTAAGGGAGTG.

### shRNA experiment

MISSION shRNA clones (PLKO.1) targeting Rgcc and control shRNA clone were purchased from Sigma-Aldrich. The lentiviruses were then packaged in 293T cells with psPAX2 and pMD2.G packaging plasmids. Viruses harvested at 48 and 72 h post transfection were combined and aliquoted for storage at –80 °C. For lentivirus transduction, LP cells cultured in 48-well plate were infected with 100 µL lentivirus containing supernatant for 1.5 days. Polybrene was added at 5 µg/mL. Lentiviral medium was then removed and refreshed with ADM1 for 2 days followed by ADM2 for 2 days. Target sequences for shRgcc clones are #1 CCTTCAGTGATGAGAAGCTGA; #2 CGAAGACTTCATTGCCGATCT.

### siRNA experiment

siRNA for gene knockdown experiment was purchased from GenePharma. Pdgfra^+^/Thy1^−^ cells from Pax7 lineage (YFP^+^) were cultured with supplement of siRNA of *Rgcc*, followed by Oil Red O staining to observe the quantity of lipid droplets. Target sequence for siRgcc is CTAAAGAGCTCGAAGACTT.

### Wnt5a ligand supplemental culture

Wnt5a ligand for culture experiment was purchased from R&D Systems. Pax7-traced YFP^+^ mouse embryos from E14.5 were sorted with Pdgfra and Thy1 antibodies and seeded on day 0. Cell culture was supplemented with 2.5 ng/mL bFGF on day 1, then Wnt5a ligand was supplemented after day 2.

### Quantification and statistical analysis

#### Transcript quantification and gene-level summarization

Kallisto^88^ was used to quantify transcripts in full-length scRNA-seq data. Index built using *Mus Musculus* transcriptome from Ensembl with addition of YFP sequence was used to quantify transcripts of individual libraries. Gene-level summarization from transcript expression was performed using R package tximport^89^.

#### Generation of single cell expression matrices

Cell Ranger 3.0.1 (10× Genomics) was used to process 3' single cell RNA-seq data. Sequencing reads were aligned to *Mus Musculus* reference genome and mapped to Ensembl GRCm38.p6. Filtered reads with valid cell barcode and UMI were used to generate gene expression matrices.

#### Dataset integration, visualization, and cell clustering

R package Seurat^90,91^ was used to handle expression matrices, visualization, and clustering. Prior to integration, cells with no YFP expression and mitochondrial gene expression percentage > 8% were removed. In addition, cells with number of genes expressed < 4000 in Smart-seq2, and counts > 15,000 in 10× were removed. A total of 5162 cells were kept for downstream analysis. Matrices were then concatenated together followed by data normalization and identification of highly variable genes. scVI^92^ was used to integrate both Smart-seq2 and 10× datasets. 2000 highly variable genes were selected to subset the concatenated matrices. The subset was passed to python environment through R package reticulate. The subset was trained with default parameters supplied with annotation to the source of the cell (Smart-seq2 or 10×). The latent representation was passed back to R for clustering and UMAP visualization.

#### Differential expression analysis

Differentially expressed genes in single-cell datasets were identified with MAST^93^. Genes with adjusted *P*-value < 0.05 were reported and used for downstream analysis. Population of test was based on number of cells in each cluster. For bulk samples, DEGs were identified using DESeq2^94^. Genes with *P*-value < 0.01 were reported or used for downstream analysis.

#### Single cell pseudotime trajectory analysis

Pseudotime trajectory analysis was performed using R package Monocle 3^53–55^. One thousand one hundred sixty-one cells in neuron and dermal pericyte clusters were removed prior to the analysis, leaving 4001 cells for analysis. Seurat object was then converted to cds object used in Monocle 3 to generate trajectory and pseudotime value.

#### Temporally expressed gene identification

Genes with expression related to pseudotime were identified by fitting expression value and pseudotime to generalized additive model (GAM). After fitting, genes with the lowest *P*-value were used for analysis. Specifically, myogenic lineage was fitted into GAM three times (EP → LM, EP → EM, EM → LM) which the 200 genes with lowest *p*-value in each fitting were selected for analysis, resulting in 363 unique genes. Therefore, 363 genes were selected in dermal and adipogenic lineage as well.

#### Gene list enrichment analysis

KEGG pathway enrichment were performed using Enrichr^95,96^, while transcription factor enrichment was performed using ChEA3^97^. Genes used were based on result from differential expression analysis. Differentially expressed genes with *P*-value < 0.01 were used for enrichment analysis.

### Reagent and resource sharing

Further information and requests for resources and reagents should be directed to and will be fulfilled by the Lead Contact Angela Wu (angelawu@ust.hk).

## Supplementary information

Supplementary Table S1

Supplementary Table S2

Supplementary Table S3

Supplementary Table S4

Supplementary Table S5

Supplementary Figures

## Data Availability

The accession number for the in-house RNA-seq data reported in this paper is GEO: GSE158887. Data generated or analyzed during this study are included in this article or available as supplementary information.
