# Supplementary material for: Cell fate determining molecular switches and signaling pathways in Pax7-expressing somitic mesoderm
Source: Cell Discov. 2022 Jun 28;8:61. doi: 10.1038/s41421-022-00407-0 (PMC9240041; doi:10.1038/s41421-022-00407-0)
Supplement: Supplementary file 1 — Supplementary Table S1 [file 41421_2022_407_MOESM1_ESM.pdf]

Supplementary figures and figure legends

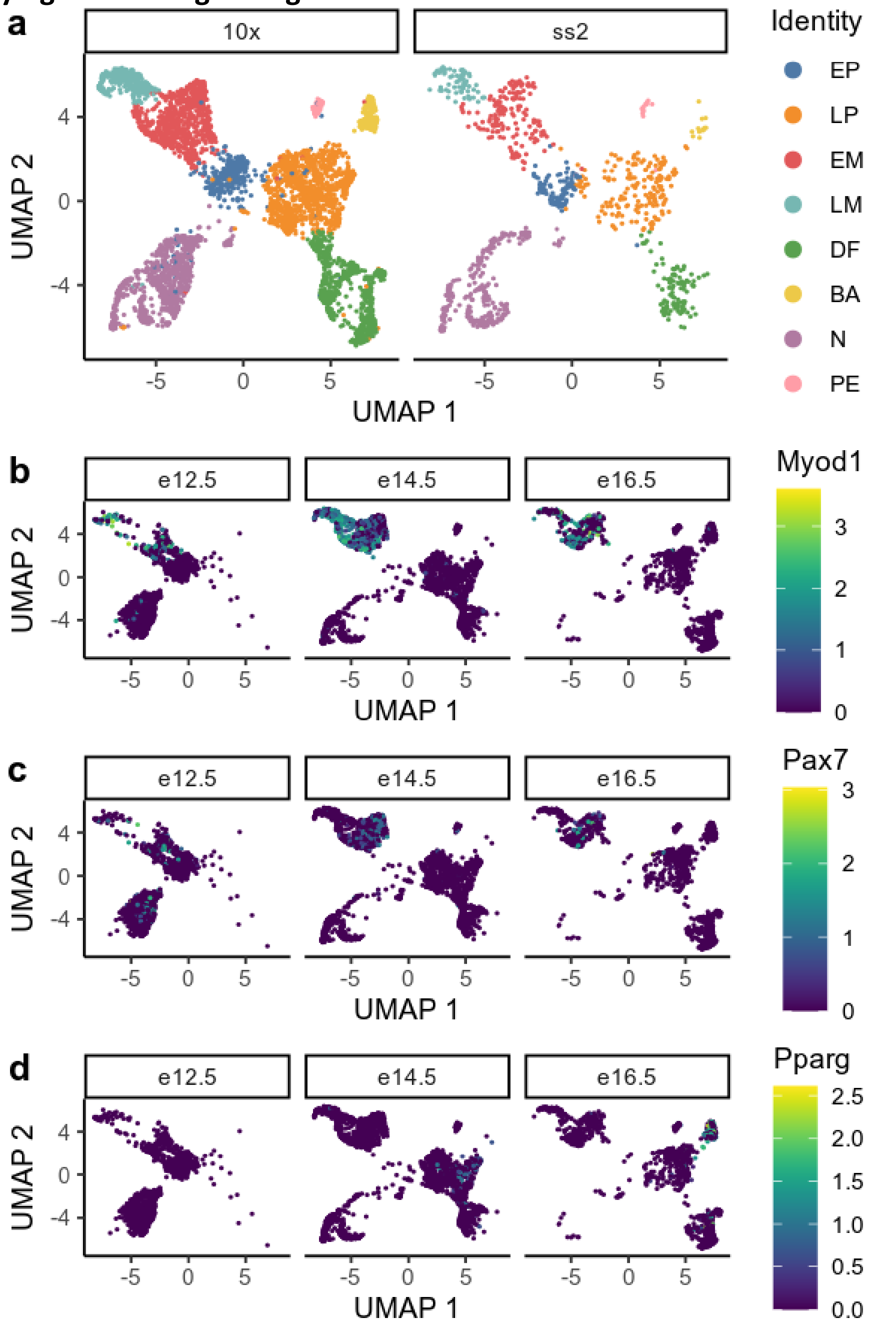

Supplementary Fig. S1: Integration result and cell type identification in Pax7 lineage, related to Fig. 1.

a UMAP visualization of integrated dataset labeled by identity and separated by library preparation method.

b-d UMAP visualization of integrated dataset colored by expression of: *Myod1* (b), *Pax7* (c), and *Pparg* (d), separated by embryonic time.

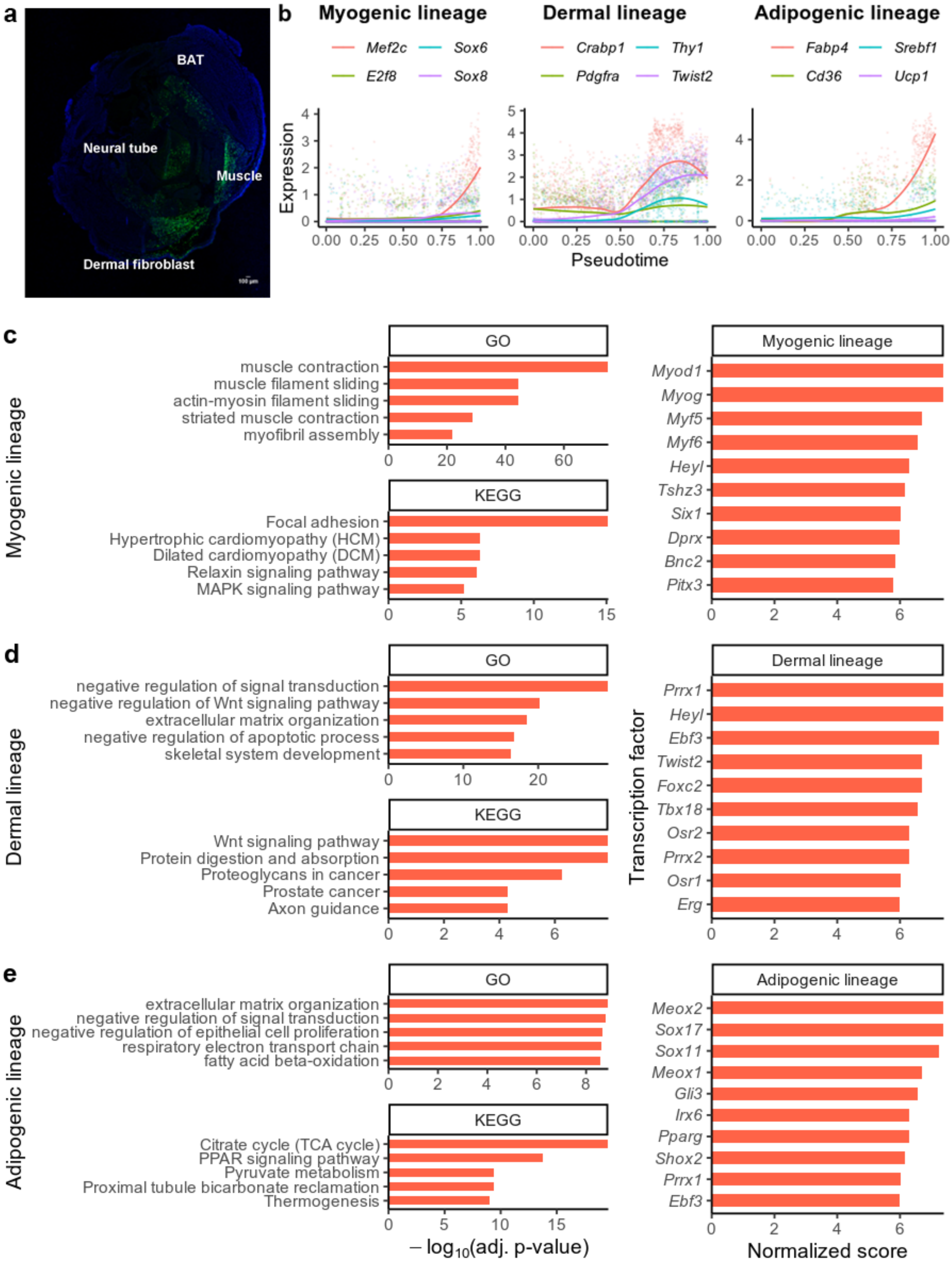

a
BAT
Neural tube
Muscle
Dermal fibroblast
b
Myogenic lineage
Mef2c
Sox6
E2f8
Sox8
Dermal lineage
Crabp1
Thy1
Pdgfra
Twist2
Adipogenic lineage
Fabp4
Srebf1
Cd36
Ucp1
Expression
Pseudotime
c
Myogenic lineage
GO
muscle contraction
muscle filament sliding
actin-myosin filament sliding
striated muscle contraction
myofibril assembly
KEGG
Focal adhesion
Hypertrophic cardiomyopathy (HCM)
Dilated cardiomyopathy (DCM)
Relaxin signaling pathway
MAPK signaling pathway
Myogenic lineage
Myod1
Myog
Myf5
Myf6
Heyl
Tshz3
Six1
Dprx
Bnc2
Pitx3
d
Dermal lineage
GO
negative regulation of signal transduction
negative regulation of Wnt signaling pathway
extracellular matrix organization
negative regulation of apoptotic process
skeletal system development
KEGG
Wnt signaling pathway
Protein digestion and absorption
Proteoglycans in cancer
Prostate cancer
Axon guidance
Dermal lineage
Transcription factor
Prrx1
Heyl
Ebf3
Twist2
Foxc2
Tbx18
Osr2
Prrx2
Osr1
Erg
e
Adipogenic lineage
GO
extracellular matrix organization
negative regulation of signal transduction
negative regulation of epithelial cell proliferation
respiratory electron transport chain
fatty acid beta-oxidation
KEGG
Citrate cycle (TCA cycle)
PPAR signaling pathway
Pyruvate metabolism
Proximal tubule bicarbonate reclamation
Thermogenesis
Adipogenic lineage
Meox2
Sox17
Sox11
Meox1
Gli3
Irx6
Pparg
Shox2
Prrx1
Ebf3
− log10(adj. p-value)
Normalized score

Supplementary Fig. S2: Pax7 lineage validation, additional temporal gene expression and gene term enrichment analysis, related to Fig. 2.

a Immunostaining of YFP on E16.5 mouse embryo cross-sections. Tamoxifen was injected at E9.5 and embryos were harvested at E16.5 and subjected to cryosection. YFP^+ cells were detected in the mesoderm regions including muscle, dermis, BAT, and also in dorsal neural tube. Scale bar: 100 µm. Nuclei were counterstained with DAPI.

b Scatter plot of temporally expressed genes to ranked pseudotime. LOESS line of expression level was shown for each gene.

c Term enrichment analysis of the myogenic lineage with GO and KEGG using EnrichR and transcription factor using ChEA3.

d Term enrichment analysis of the dermal lineage.

e Term enrichment analysis of the adipogenic lineage.

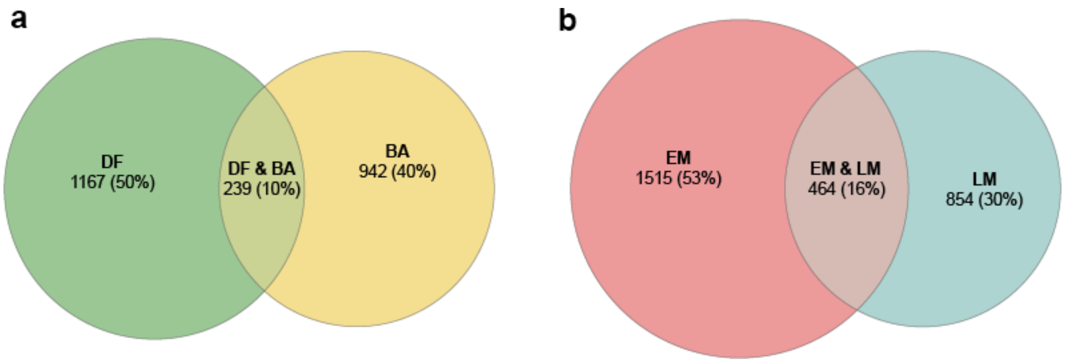

Supplementary Fig. S3: Venn diagram of highly variable genes, related to Fig. 3.
a Venn diagram of high variable genes between DF and BA.
b Venn diagram of high variable genes between EM and LM.

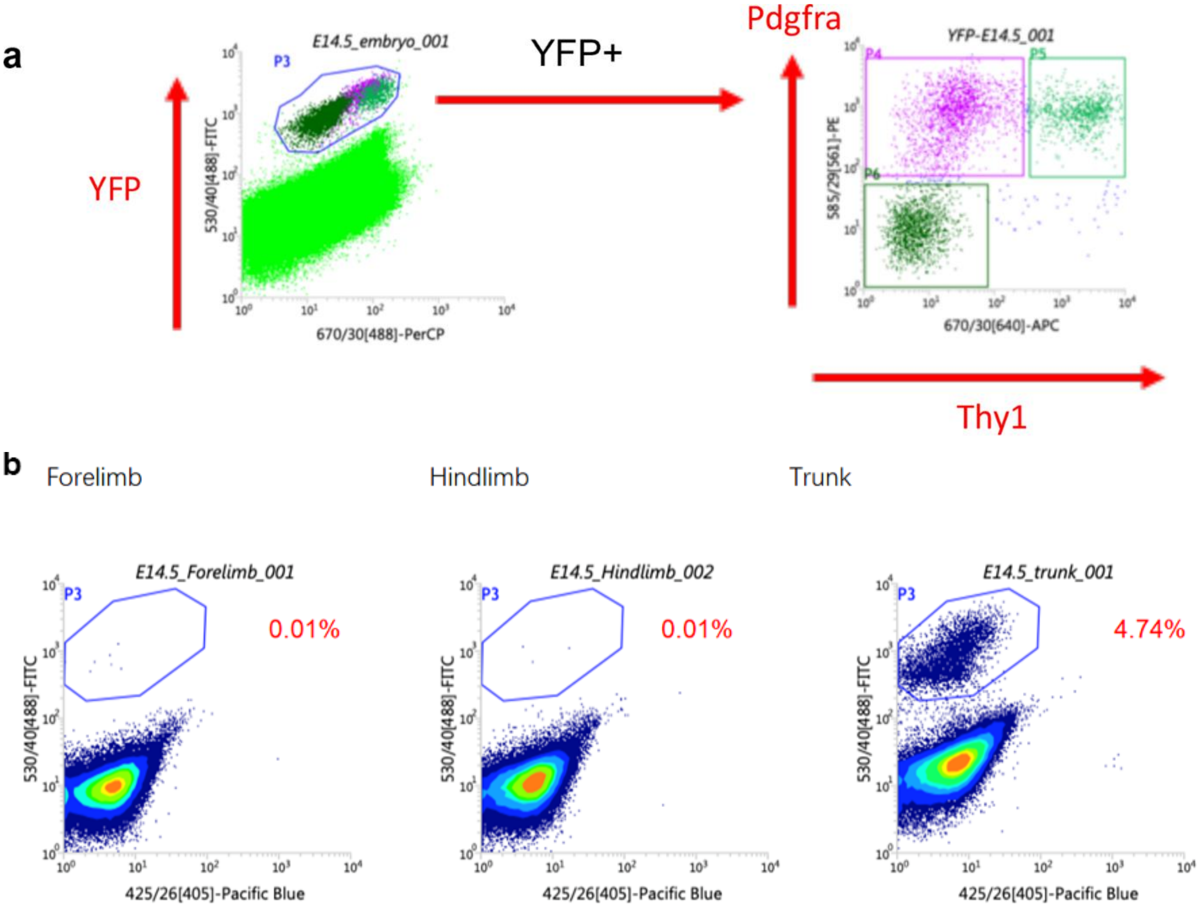

Supplementary Fig. S4: Sorting strategy validation by FACS, related to Fig. 4.
a FACS plot of Pax7-traced mouse embryos at E14.5. Embryos were labeled by TMX at E9.5 and harvested at E14.5.
b FACS plot of forelimb, hindlimb and trunk of E14.5 Pax7-traced mouse embryos. Few YFP+ cells were observed in both forelimb and hindlimb, while 4.74% of cells from trunk were YFP+ (percentage of detected events under area P3).

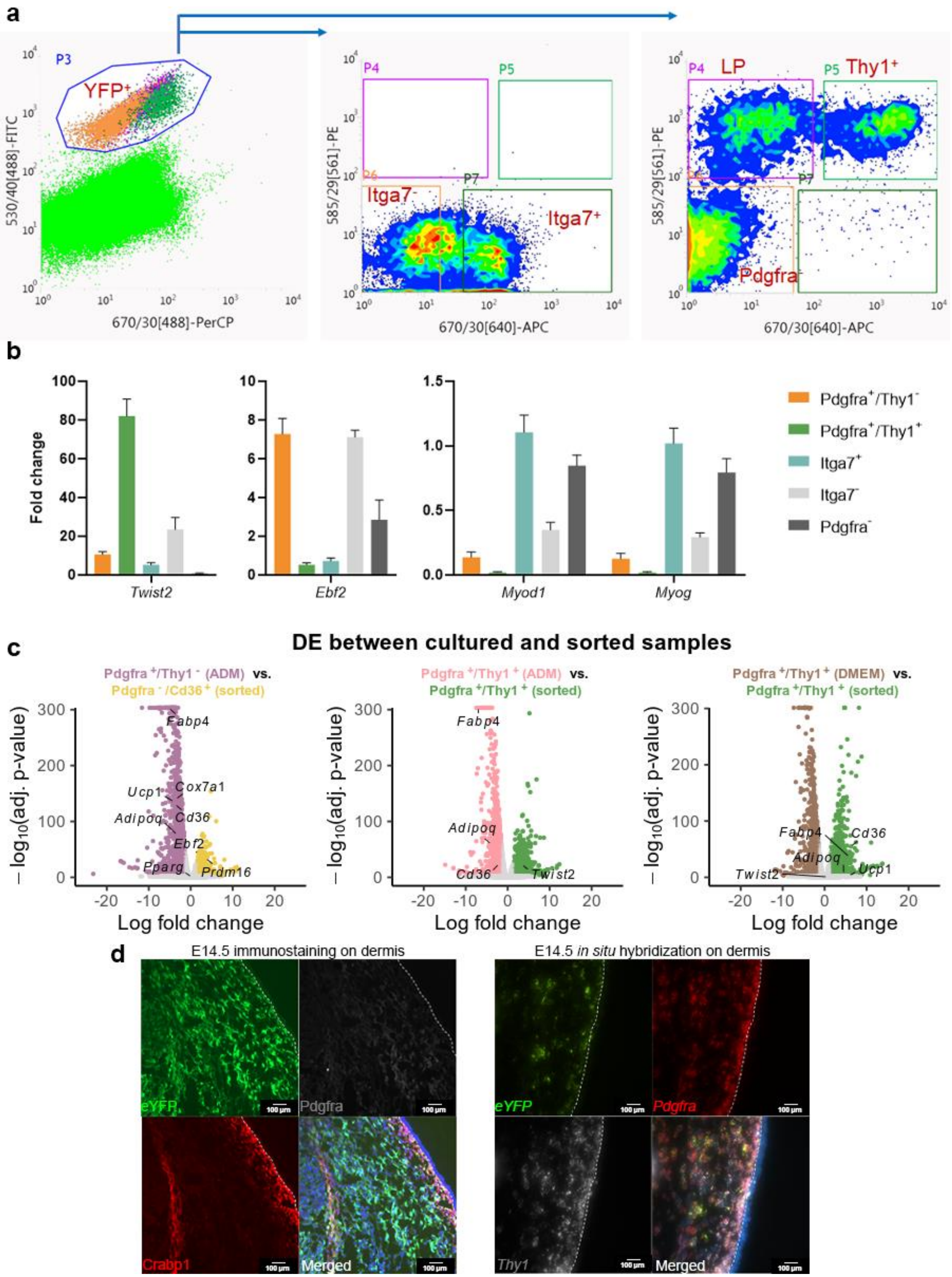
a
P3
YFP+
530/40[488]-FITC
670/30[488]-PerCP
P4
P5
P6
P7
Itga7-
Itga7+
585/29[561]-PE
670/30[640]-APC
LP
Thy1+
Pdgfra-
b
Fold change
Twist2
Ebf2
Myod1
Myog
Pdgfra+/Thy1-
Pdgfra+/Thy1+
Itga7+
Itga7-
Pdgfra-
c
DE between cultured and sorted samples
Pdgfra+/Thy1- (ADM) vs. Pdgfra-/Cd36+ (sorted)
Pdgfra+/Thy1+ (ADM) vs. Pdgfra+/Thy1+ (sorted)
Pdgfra+/Thy1+ (DMEM) vs. Pdgfra+/Thy1+ (sorted)
− log10(adj. p-value)
Log fold change
Fabp4
Ucp1
Cox7a1
Adipoq
Cd36
Ebf2
Pparg
Prdm16
Twist2
d
E14.5 immunostaining on dermis
E14.5 in situ hybridization on dermis
eYFP
Pdgfra
Crabp1
Merged
Thy1
100 µm

Supplementary Fig. S5: Gene expression validation in cells enriched by sorting strategy, related to Fig. 5.

a FACS plot of E14.5 Pax7 lineage-traced mouse embryos, with YFP signal indicated by y-axis. YFP^{+} cells were further divided into subgroups by surface markers Thy1, Pdgfra, and Itga7.

b Bar chart showing RT-qPCR of *Twist2*, *Ebf2*, *Myod1* and *Myog* expressions in samples isolated by surface markers described in a. Expression of first replicate in $Pdgfra^{-}$ group were set as 1, expression levels in other group were normalized as fold change over normalized $Pdgfra^{-}$ group. Values are mean ± SEM, n = 3.

c Volcano plots of differentially expressed genes between sorted and cultured samples. Genes with adjusted p-value < 0.01 were colored by orange (log-fold change > 1.5) or blue (log-fold change < -1.5), whereas ≥ 0.01 were colored as grey. Lineage markers and surface markers were labelled adjacent to corresponding dots.

d Section staining (left) and *in situ* hybridization (right) of E14.5 mouse embryo showing dermis. eYFP, Pdgfra and Crabp1 signals, as well as *eYFP*, *Pdgfra* and *Thy1* were observed at dermis while only DAPI was observed at epidermis.

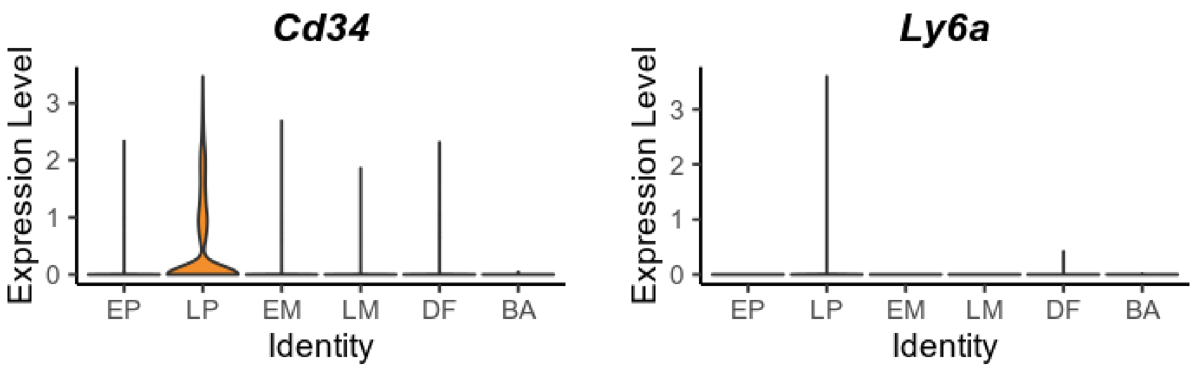

Supplementary Fig. S6: Violin plot of *Cd34* and *Ly6a* (Sca-1) expression in LP
